# Dominance of Deleterious Alleles Controls the Response to a Population Bottleneck

**DOI:** 10.1371/journal.pgen.1005436

**Published:** 2015-08-28

**Authors:** Daniel J. Balick, Ron Do, Christopher A. Cassa, David Reich, Shamil R. Sunyaev

**Affiliations:** 1 Division of Genetics, Brigham and Women’s Hospital, Boston, Massachusetts, United States of America; 2 Department of Medicine, Harvard Medical School, Boston, Massachusetts, United States of America; 3 Broad Institute of Harvard and MIT, Cambridge, Massachusetts, United States of America; 4 The Charles Bronfman Institute for Personalized Medicine, Icahn School of Medicine at Mount Sinai, New York, New York, United States of America; 5 The Center for Statistical Genetics, Icahn School of Medicine at Mount Sinai, New York, New York, United States of America; 6 Department of Genetics and Genomic Sciences, Icahn School of Medicine at Mount Sinai, New York, New York, United States of America; 7 Howard Hughes Medical Institute, Harvard Medical School, Boston, Massachusetts, United States of America; University of California Davis, UNITED STATES

## Abstract

Population bottlenecks followed by re-expansions have been common throughout history of many populations. The response of alleles under selection to such demographic perturbations has been a subject of great interest in population genetics. On the basis of theoretical analysis and computer simulations, we suggest that this response qualitatively depends on dominance. The number of dominant or additive deleterious alleles per haploid genome is expected to be slightly increased following the bottleneck and re-expansion. In contrast, the number of completely or partially recessive alleles should be sharply reduced. Changes of population size expose differences between recessive and additive selection, potentially providing insight into the prevalence of dominance in natural populations. Specifically, we use a simple statistic, $B_{R}\equiv \sum x_{i}^{pop1}/\sum x_{j}^{pop2}$, where *x*_*i*_ represents the derived allele frequency, to compare the number of mutations in different populations, and detail its functional dependence on the strength of selection and the intensity of the population bottleneck. We also provide empirical evidence showing that gene sets associated with autosomal recessive disease in humans may have a *B*_*R*_ indicative of recessive selection. Together, these theoretical predictions and empirical observations show that complex demographic history may facilitate rather than impede inference of parameters of natural selection.

## Author Summary

Dominance has played a central role in classical genetics since its inception. However, the effect of dominance introduces substantial technical complications into theoretical models describing dynamics of alleles in populations. As a result, dominance is often ignored in population genetic models. Statistical tests for selection built on these models do not discriminate between recessive and additive alleles. We show that historical changes in population size can provide a way to differentiate between recessive and additive selection. Our analysis compares two sub-populations with different demographic histories. History of our own species provides plenty of examples of sub-populations that went through population bottlenecks followed by re-expansions. We show that demographic differences, which generally complicate the analysis, can instead aid in the inference of features of natural selection.

## Introduction

In diploid organisms, the fitness effect of an allele, or a group of alleles, can be categorized as additive, dominant or recessive, or as part of a more general epistatic network. A large body of existing work is devoted to the development of statistical methods for the detection and quantification of selection using DNA sequencing data, including comparative genomics and the sequencing of population samples [1–3]. However, much less progress has been made toward developing methods to identify the mode of selection as additive, recessive or dominant. Substantial experimental work in the last 50 years has been devoted to identifying the average dominance coefficient in model organisms, often with disagreement between different studies and techniques [4, 5]. These studies, in an attempt to identify the relationship between dominance coefficients and selective effects, largely focus on mutation accumulation experiments and subsequent laboratory propagation, determining dominance coefficients from the viability of crosses [4, 6]. At least one study attempts to determine the relationship between dominance coefficient and selective effect from natural populations, propagating crosses directly from wild-type samples, however the methodology relies on the often inapplicable assumption of mutation-selection balance [7]. A particularly useful overview of various techniques and studies can be found in [8], with some more modern techniques described in [9]. Additionally, more recent work taking advantage of a large amount of yeast knockout data has made progress towards quantifying the distribution of dominance effects (restricted to the discussion of nonsense mutations), with emphasis on the variance and skew of this distribution [10, 11].

Despite these substantial steps forward, all of the methods employed rely on the ability to rapidly breed laboratory-friendly organisms, either for the purposes of mutation accumulation or production of homozygotes and heterozygotes through crosses. Unfortunately, such techniques are infeasible when dealing with long-lived macroscopic organisms, particularly in the case of humans. In the present work, we hope to provide steps towards the development of techniques applicable to natural populations of such organisms by making use of naturally occurring demographic events and describing the dynamic response of populations to such events.

The genetics of model organisms and of human disease provide plenty of anecdotal evidence in favor of the general importance of dominance [12]. Although genome-wide association studies suggest that alleles of small effects involved in human complex traits frequently act additively, estimation of genetic variance components from large pedigrees suggests a substantial role for dominance in a number of human quantitative traits; LDL cholesterol levels, for example, have a substantial dominance component, as shown in [13]. Alleles of large effects involved in human Mendelian diseases often behave similarly to large effect (and even lethal) spontaneous and induced mutations in model organisms, such as mouse, zebrafish, or flies, that are frequently recessive [4, 14]. In spite of these observations, the role of dominance in population genetic variation and evolution remains largely unexplored in the majority of diploid species and no formal statistical framework is currently available to identify dominance coefficients in natural populations deviating from mutation-selection balance.

A number of theoretical studies suggested that demographic processes associated with the increase in variance of allele frequency distribution result in a more efficient removal of recessive deleterious alleles [15–18]. Such demographic scenarios include population bottlenecks, population subdivision, range expansion, and inbreeding. Increase in the variance of allele frequency distribution during a bottleneck can be characterized by inbreeding coefficient (even in case of a panmictic population). For structured populations, the increase in variance is characterized by *F*_*ST*_. Substantial theoretical work and associated experimental studies explored the removal of recessive variants due to increased inbreeding coefficient during sustained population bottlenecks [19–22]. Additionally, several studies note that bottlenecks have a strong effect on nonadditive variation, specifically loci with epistatic interactions [19, 23–30]. To complement these analyses, we focus on genetic variation in panmictic populations that experienced a population bottleneck and subsequent re-expansion, similar to the scenario recently analyzed in [30]. Using a combination of theoretical analysis and computer simulations, we demonstrate that recessive selection can be qualitatively distinguished from additive selection in populations that recently recovered from a temporary bottleneck, and detail the dynamics of the average number of mutations per haploid.

An important study by Kirkpatrick and Jarne [31] qualitatively described how, perhaps counterintuitively, the number of deleterious recessive alleles per haploid genome is transiently reduced after re-expansion following a population bottleneck, while the number of additively or dominantly acting alleles is increased. We focus on this insight and quantitatively extend the analysis of these dynamics to show that, in spite of a well-documented increase in the frequency of some recessively acting variants in founder populations, the average number of deleterious recessive alleles (with dominance coefficient *h* ≪ 0.5) carried by an individual is reduced as a consequence of the bottleneck. With the growing availability of DNA sequencing data in multiple populations, these results demonstrate the potential to directly evaluate the role of dominance, either on a whole genome level, or in specific categories of genes.

Population bottlenecks are a common feature in the history of many human populations. For example, the “Out of Africa” bottleneck involved the ancestors of many present-day human populations. Numerous recent bottlenecks affected, among others, the well studied populations of Finland and Iceland. More generally, bottlenecks followed by expansions are standard features in the recent evolution of most domesticated organisms, including an analogous “Out of Africa” event in *Drosophila melanogaster* [32], highlighting the ubiquity of these events in natural populations. We suggest that complex demographic history may assist rather than complicate statistical inference of selection in population genetics.

**Fig 1:**
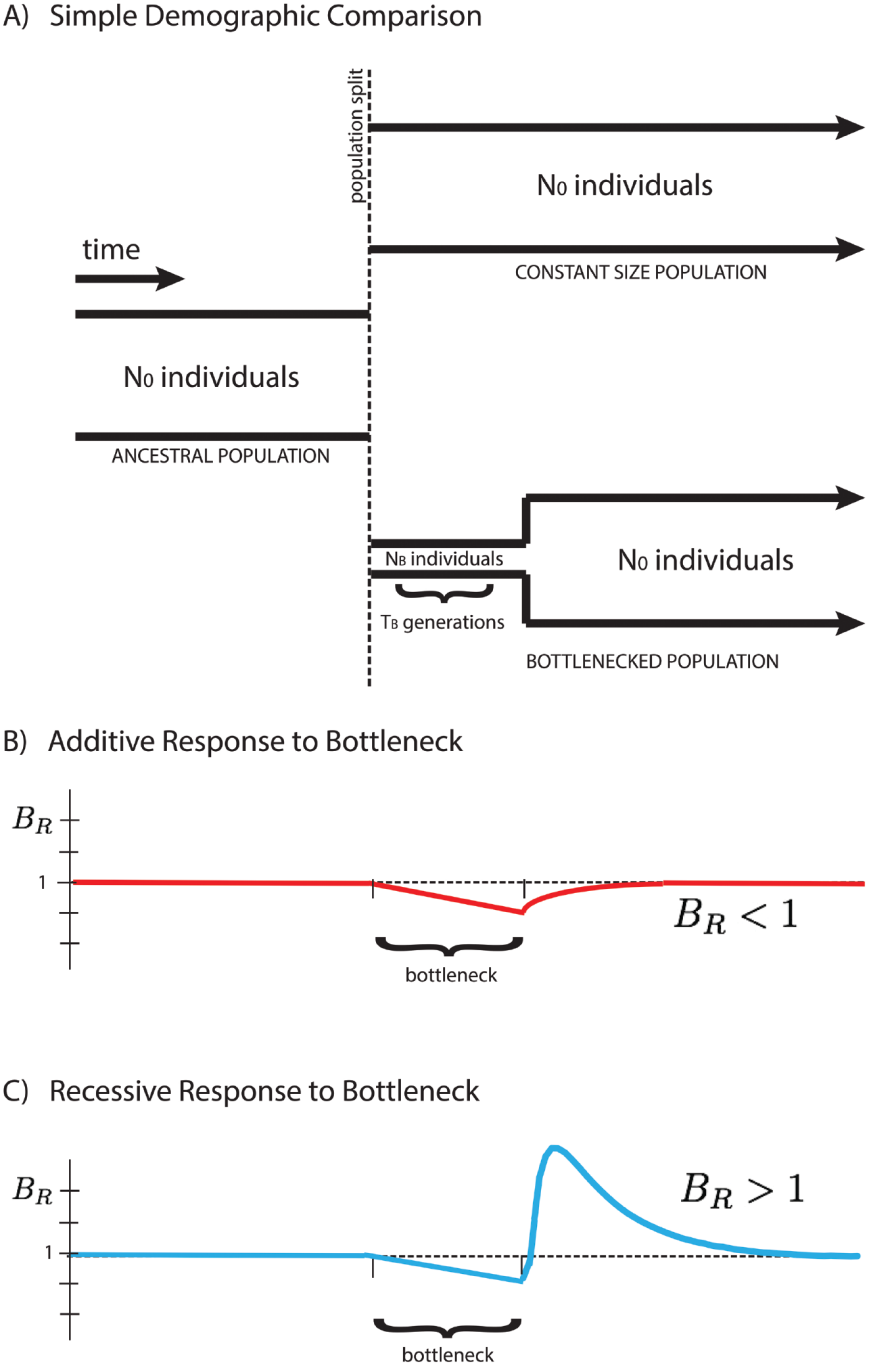
Response of the *B*_*R*_ statistic for additive and recessive variation. A schematic representation of two populations is presented above (**A**). Initially a single population prior to the bottleneck event, the populations split and have distinct demographic profiles. The equilibrium population maintains a constant size for easy comparison to the founded population. The latter drastically reduces its population size to *N*_*B*_ for a short time *T*_*B*_ during the founder’s event. Our statistical comparison between populations $B_{R}=\frac{{\langle x\rangle }_{eq}}{{\langle x\rangle }_{founded}}$ is represented here for cases of purely additive (**B**) and purely recessive (**C**) variation. The statistic *B*_*R*_ > 1 for recessive variation (dominance coefficient *h* = 0) and *B*_*R*_ < 1 for additive variation (*h* = 1/2), providing a simple indicator for the primary mode of selection of polymorphic alleles in the populations.

Here we focus on a comparison between two populations that recently split, after which their demographic histories diverged, one exhibiting a founder’s event (a population bottleneck followed by subsequent re-expansion), and the other maintaining a fixed population size. We analyze their accumulated differences to shed light on the type of selection dominating the dynamics of deleterious alleles, and show that the average number of mutations per individual, 〈*x*〉, is dependent on the mode of selection characterized by the average dominance coefficient, *h*. We introduce a measure *B*_*R*_ (the “burden ratio” defined below) that is the ratio of per-haploid deleterious allele accumulation in the two populations. This potentially allows for the qualitative distinction between predominantly additive selection (*h* ≈ 0.5), where mutations accumulate due to relaxed selection during a bottleneck, resulting in *B*_*R*_ < 1, and predominantly recessive selection (*h* ≪ 0.5), where homozygous deleterious mutations are purged from the population after re-expansion from the bottleneck, resulting in *B*_*R*_ > 1, as shown in Fig 1.

For qualitative demonstration and development of intuition, the analysis assumes strictly additive and strictly recessive selection with a highly idealized demography. However, this behavior is not restricted to the simplified demographic model presented in this paper, but rather suggests a quite generic qualitative signature for the presence of recessive (or near-recessive) selection in comparison between two populations, one of which experienced a bottleneck event. Additionally, our simulations suggest the potential to distinguish between partially recessive and additive alleles, as the change in the qualitative behavior of *B*_*R*_ occurs at intermediate values of the dominance coefficient, *h*. The temporal dependence of the “critical dominance coefficient”, *h*_*c*_, describing the boundary between *B*_*R*_ > 1 and *B*_*R*_ < 1, as well as the sensitivity to partial recessivity, is discussed in the S1 Text.

To ask whether the behavior of the *B*_*R*_ statistic is consistent with the dynamics of recessive selection in natural populations, we perform a statistical analysis of genes annotated in the literature as causing autosomal recessive (AR) disease. We use the “Out of Africa” event to differentiate between variation in African and European populations, potentially allowing for the identification of recessive selection in natural human populations. We find that sets of AR disease genes show a statistically significant deviation from neutrality, with *B*_*R*_ > 1. This suggests that at least some disease-associated genes with autosomal recessive mode of inheritance may be under recessive selection. Although this observation is not surprising, it is nontrivial, as disease genes could be neutral, highly pleiotropic, or contain variants with different modes of inheritance. This analysis demonstrates the potential to use our methodology to identify sets of genes under predominantly recessive selection.

## Results

### Model

We work with a simple demography described by an ancestral population of *N*_0_ individuals that splits into two subpopulations, one with population size *N*_0_ equal to the initial population size (“equilibrium”), and one with reduced bottleneck population size *N*_*B*_ (“founded”). The latter population persists at this size for *T*_*B*_ generations before instantaneously re-expanding to the initial population size *N*_0_, as shown in Fig 1. Time *t* is measured after the re-expansion from the bottleneck, as we are interested in the dynamics during this period. Quantities measured in the equilibrium population, and equivalently prior to the split, are denoted with a subscript “_0_”. We consider only deleterious mutations with average selective effect of magnitude *s* > 0, such that *s* represents the strength of deleterious selection. Extensions of this analysis to a full distribution of selective effects can be found in the S1 Text. The initial population is in a quasi-steady state with 2*N*_0_ *U*_*d*_ deleterious alleles introduced into the population with a one-way mutation rate *U*_*d*_ per haploid individual per generation and rare fixation of deleterious alleles. In the absence of back-mutations, the population is not strictly in static equilibrium, however, this approximation is reasonable when the back-mutation rate and average derived allele frequencies are relatively low. In approximate equilibrium, the site frequency spectrum (SFS), denoted *ϕ*(*x*), for polymorphic alleles is given by Kimura [33].

$$\begin{array}{c} \phi_{eq}\left(x\right)=4NU_{d}\frac{e^{-4Nshx-2Ns\left(1-2h\right)x^{2}}}{x(1-x)}[1-\frac{\int_{0}^{x}dy\;e^{4Nshy+2Ns\left(1-2h\right)y^{2}}}{\int_{0}^{1}dy\;e^{4Nshy+2Ns\left(1-2h\right)y^{2}}}] \end{array}$$ (1)

Here *h* ≥ 0 is the dominance coefficient for deleterious mutations, where *h* = 1/2 corresponds to a purely additive set of alleles, and *h* = 0 corresponds to the purely recessive case. For the present analysis, we primarily focus on these two limits, contrasting their effects on the genetic diversity. An expanded discussion of the treatment of intermediate dominance coefficients can be found in the S1 Text. The solution represents a mutation-selection-drift balance in which new mutations are exactly compensated for by the purging of currently polymorphic alleles by both selection and extinction due to stochastic drift. In this way, an approximately static number of polymorphic alleles exists in the population at any given time.

### Population dynamics

As noted above, a qualitative insight on the effect of the bottleneck on recessive variation was previously obtained by noting that the expected change in frequency of recessive allele is accelerated due to the increased variance of allele frequencies (inbreeding coefficient). We offer a different approach and attempt to quantitatively describe the difference in dynamics between additive and recessive variation.

We follow the expected number of mutations per chromosome in the population, noting that it is simply the first moment of SFS.

$$\begin{array}{c} \langle x\rangle =\int x\;\phi (x) \end{array}$$ (2)

When multiplied by *s*, this is the effective “mutation load” of each individual in the additive case, but in the case of purely recessive selection this is not proportional to the fitness, as selection acts only on homozygotes. We refer to this statistic generally as the “mutation burden” to avoid assumption of any given mode of selection. As described below, comparison between the mutation burden in the equilibrium and founded populations in the form of the “burden ratio”, *B*_*R*_, may prove useful in the identification of sets of alleles under recessive selection.

$$\begin{array}{c} B_{R}\equiv \frac{{\langle x\rangle }_{eq}}{{\langle x\rangle }_{founded}}=\{\begin{array}{cc} <1 & \text{for}\;\text{additive}\;\text{selection}\\ >1 & \text{for}\;\text{recessive}\;\text{selection} \end{array} \end{array}$$ (3)

To gain intuition for this qualitative difference, we work to quantitatively understand the population dynamics in a simple demography, first for purely additive selection, and then for purely recessive selection for comparison.

#### Additive selection and response to a bottleneck

The initial site frequency spectrum $\phi_{0}^{A}(x)$ for purely additive alleles is given by Eq (1) with *h* = 1/2.

$$\begin{array}{c} \phi_{0}^{A}\left(x\right)=\frac{\theta_{0}}{x(1-x)}\frac{1-e^{2N_{0}s\left(1-x\right)}}{1-e^{2N_{0}s}} \end{array}$$ (4)

Here *θ*_0_ = 4*N*_0_ *U*_*d*_. In the deterministic limit, when 2*N*_0_ *s* ≫ 1, the SFS rapidly decays as *x* → 1 simplifying the functional form [34]. We approximately compute the initial mutation burden as follows.

$$\begin{array}{c} {\langle x\rangle }_{0}\approx \theta_{0}\int_{0}^{1}x\;\frac{e^{-2N_{0}sx}}{x}\approx \frac{2U_{d}}{s} \end{array}$$ (5)

This describes the deterministic mutation-selection balance for mutations under strong selection. Now we deviate from equilibrium by reducing the population size to 2*N*_*B*_ chromosomes, representing a population bottleneck. The effect that a bottleneck has on the site frequency spectrum is twofold: a fraction of alleles are removed from the population due to increased random drift, and the mean of the remaining alleles occurs at higher frequency. The dynamics of the distribution *ϕ*(*x*, *t*) during such a change in demography can be computed from Kolmogorov’s forward equation, as detailed in the S1 Text. The first moment of the distribution, the mutation burden, follows the temporal dynamics derived from summing the Kolmogorov equation over all alleles in the genome, and takes the following form.

$$\begin{array}{c} \partial_{t}\langle x\rangle \approx U_{d}-\frac{s}{2}(\langle x\rangle -\langle x^{2}\rangle ) \end{array}$$ (6)

As discussed in [35, 36], the burden of additive mutations is not directly affected by drift, as the drift term vanishes from the dynamics of the first moment, however the dependence on the second moment introduces an indirect dependence on drift. In the strong selection regime, in the limit where 〈*x* ^2^〉 ≪ 〈*x*〉, extinction of some alleles is exactly compensated for by an increase in the frequency of other alleles. This is true in the equilibrium distribution prior to the bottleneck when *N*_0_ *s* ≫ 1, where ${\langle x\rangle }_{0}∼O\left(U_{d}/s\right)$ and $\langle x_{0}^{2}\rangle ∼𝒪\left(U_{d}/(N_{0}s^{2})\right)$, as can be computed directly from Eq (4). During the bottleneck the mutation burden 〈*x*〉 monotonically increases; the second moment 〈*x* ^2^〉 increases, as well, reaching a maximum value in the case of a long bottleneck where it has re-equilibrated and scales as $\langle x^{2}\rangle ∼O\left(U_{d}/(N_{B}s^{2})\right)$. Provided *N*_*B*_ *s* ≫ 1, the second moment is guaranteed to be subdominant to the first moment, such that Eq (6) is well approximated by $\partial_{t}\langle x\rangle \approx U_{d}-\frac{s}{2}\langle x\rangle$ in the strong selection limit with the well known solution $\langle x\rangle \approx \frac{2U_{d}}{s}$. For a finite duration bottleneck of *T*_*B*_ generations, the population immediately recovers and remains in mutation-selection balance throughout the bottleneck with final burden $\langle x(T_{B})\rangle \approx \frac{2U_{d}}{s}$. After instantaneous re-expansion to the initial population size, the dynamics of the distribution *ϕ*(*x*) are completely analogous to those inside the bottleneck in this limit, such that the mutation burden never deviates during the demographic perturbation.

In the opposite limit of completely relaxed selection during the bottleneck, the dynamics of the mutation burden are completely driven by the influx of new mutations. For a bottleneck with duration *T*_*B*_ generations, the net effect of mutation accumulation due to relaxed selection is given simply by the following expression.

$$\begin{array}{c} \langle x\left(T_{B}\right)\rangle \approx {\langle x\rangle }_{0}+U_{d}T_{B} \end{array}$$ (7)

Additionally, one can show that the second non-central moment gains an analogous contribution in addition to the net effect of drift.

$$\begin{array}{c} \langle x^{2}\left(T_{B}\right)\rangle \approx {\langle x^{2}\rangle }_{0}+I_{B}{\langle x\rangle }_{0}+U_{d}I_{B} \end{array}$$ (8)

Here we have expressed the second moment as a function of the bottleneck intensity $I_{B}\equiv \frac{T_{B}}{2N_{B}}$. Immediately after re-expansion from the bottleneck, selection is again efficient, so that the dynamics are completely described by Eq (6). Although the second moment is increased due to relaxed selection during the bottleneck, we find that this increase is negligible in comparison to the direct accumulation of the first moment provided that *I*_*B*_ ≪ 1. As a result, the primary effect of the bottleneck in this limit is to accrue new mutations that are subsequently purged when selection is again efficient in the re-expanded population. The dynamics for the two limiting cases can be summarized as follows.

$$\begin{array}{c} {\langle x\left(t\right)\rangle }_{founded}\approx \{\begin{array}{cc} \frac{2U_{d}}{s} & \;\text{for}\;\;\;2N_{B}s\gg 1\\ \frac{2U_{d}}{s}+U_{d}T_{B}e^{-\frac{st}{2}} & \;\text{for}\;\;\;2N_{B}s\ll 1,\;\;\;I_{B}\ll 1 \end{array} \end{array}$$ (9)

Here *T*_*B*_ represents the duration of the bottleneck, and *t* represents the time after re-expansion from the bottleneck. The top result is for the deterministic strong selection limit, and the bottom result is for the case of completely relaxed selection such that during the bottleneck the dynamics are effectively neutral. We note that 〈*x*〉_*founded*_ ≥ 〈*x*〉_*eq*_ at all times in both limiting cases, and asymptotically decays to the equilibrium frequency on a timescale given by the strength of selection of the accumulated deleterious mutations. In the case of a single-generation bottleneck, we find that the mutation burden is only slightly shifted even if selection is fully relaxed, resulting in effectively no observable change at either limit. Our statistical measure, the burden ratio *B*_*R*_, in the additive case can be written approximately as follows.

$$B_{R}{}^{additive}\left(t\right)=\frac{{\langle x\rangle }_{eq}}{{\langle x\rangle }_{founded}}\approx \{\begin{array}{ll} 1 & \text{for}\;\;\;2N_{B}s\gg 1\\ {\left(1+\frac{sT_{B}}{2}e^{-\frac{st}{2}}\right)}^{-1}\leq \;1 & \text{for}\;\;\;2N_{B}s\ll 1,\;I_{B}\ll 1 \end{array}$$ (10)

We note that the mutation burden in each population is proportional to the mutation rate, such that mutation rates cancel as long as they are the same in both populations leaving *B*_*R*_ independent of mutation rate.

As we will see in the following sections, recessive selection results in a depleted mutation burden with corresponding values *B*_*R*_ > 1, proving a contrast to the additive scenario and is thus a signature of recessivity.

#### Recessive selection and dynamics of the mutation burden

Prior to the bottleneck, the initial site frequency spectrum for alleles under recessive selection is given by the *h* = 0 limit of Eq (1).

$$\begin{array}{c} \phi_{0}^{R}\left(x\right)=\theta_{0}\frac{e^{-2N_{0}sx^{2}}}{x(1-x)}[1-\frac{\int_{0}^{x}dy\;e^{2N_{0}sy^{2}}}{\int_{0}^{1}dy\;e^{2N_{0}sy^{2}}}] \end{array}$$ (11)

At low frequencies with $x<\sqrt{4N_{0}s}$ the spectrum decays more slowly than in the additive case, representing alleles protected from recessive selection by existing primarily in heterozygous form. In contrast, at high frequencies the spectrum decays faster than the additive exponential decay, falling off as *e* ^−2*N*_0_*sx*^2^^.

#### Single-generation population bottlenecks

First, we restrict our analysis to a single-generation bottleneck with intensity *I*_*B*_ = 1/2*N*_*B*_, as this provides insight into the non-equilibrium response of the frequency spectrum to a downsampling event. Later, we extend our analysis to finite bottlenecks that persist for *T*_*B*_ generations, with total intensity *I*_*B*_ = *T*_*B*_/2*N*_*B*_. We represent the increase in drift due to a single-generation bottleneck by downsampling. During this time step, *N*_*B*_ diploid individuals are chosen at random from the initial larger population of *N*_0_ individuals.

ϕB(k,tB=0)=(2NBk)∫dy(1-y)2NB-k(y)kϕ0(y) (12)

Binomial sampling gives the distribution *ϕ*_*B*_ of deleterious alleles with frequency *x* = *k*/2*N*_*B*_. There is a loss of allelic variation due to the bottleneck, corresponding to the *k* = 0 term in Eq (12).

Re-expansion is modeled as up-sampling the distribution *ϕ*_*B*_(*x*) from *N*_*B*_ to *N*_0_ diploid individuals, which has a negligible effect on the first and second moments of the distribution. As a result of drift to higher frequencies during the bottleneck, much of the existing variation appears in homozygous form immediately after the increase in population size. These individuals are rapidly selected out of the population, driving high frequency alleles to lower frequencies on a very short time scale, as was initially described in [31]. Since drift is once again suppressed, selection becomes far more efficient, particularly for alleles of large selective effect.

The time evolution of *ϕ* after the bottleneck is given by the forward Kolmogorov equation for recessive selection (see S1 Text). The mutation burden follows the time dependence,

$$\begin{array}{c} \partial_{t}\langle x\left(t\right)\rangle \approx U_{d}-s\langle x{\left(t\right)}^{2}\rangle . \end{array}$$ (13)

Here we suppress a selection term proportional to 〈*x* ^3^〉 of $𝒪(1/\sqrt{Ns})$ in analogy to the additive case. Since recessive selection depends quadratically, rather than linearly, on the allele frequency, the increased variance of the distribution drives the motion of the mutation burden. Alleles with frequency $x>\sqrt{1/2N_{0}}$ appear in homozygous form and are rapidly pushed down to lower frequencies. This happens on a time scale of order *s* ^−1/2^ and effectively reduces the variance, slowing the decrease in the mutation burden 〈*x*〉. New mutations introduced during this period slowly drift to appreciable frequencies, replacing those lost in the bottleneck. This process is drift controlled, rather than selection controlled, and thus occurs on a time scale of $O\left(2N_{0}\right)$ generations. As a result, the mutation burden quickly decreases due to selection immediately after the bottleneck until it slows to a stop, and then gradually increases as the population accumulates new mutations and re-equilibrates.

A minimum in the mutation burden 〈*x*(*t*)〉_*founded*_ occurs when the time derivative vanishes. This corresponds to a characteristic time scale associated with the selective effect *s*, where our statistical measure $B_{R}=\frac{{\langle x\rangle }_{eq}}{{\langle x\rangle }_{founded}}$ is maximized. Since this time scale is shorter than the time scale of drift, we can imagine rescaling time by the effective population size 2*N*_0_ and then working in the perturbative regime *t*/2*N*_0_ ≪ 1. This allows us to Taylor expand near the re-expansion time *t* = 0 to understand the motion of the mutation burden at times soon after the bottleneck.

$$\begin{array}{c} \partial_{t}\langle x\left(t\right)\rangle \approx U_{d}-s[\langle x{\left(t\right)}^{2}\rangle {|}_{t=0}+t\partial_{t}\langle x{\left(t\right)}^{2}\rangle {{|}_{t=0}+\frac{t^{2}}{2}\partial_{t}^{2}\langle x{\left(t\right)}^{2}\rangle |}_{t=0}+O\left(t^{3}\right)] \end{array}$$ (14)

To understand the time dependence of 〈*x* ^2^〉, specifically the time derivative, we analyze the higher moments in the same fashion as employed for the first moment in Eq (13). All relevant moments are computed in the S1 Text and we note sufficient convergence to validate this expansion. This allows for the re-expression of Eq (14) to second order in *t* in terms of the first three moments of the site frequency spectrum immediately after re-expansion. The moments of the post-bottleneck initial distribution can be written in terms of the initial equilibrium distribution using the integral form given in Eq (12). Details of this calculation appear in the S1 Text. In the strong selection limit 2*N*_0_ *s* ≫ 1 these initial equilibrium moments are readily approximated by standard convolutions of a polynomial with a Gaussian. Suppressing subdominant contributions in the limit $N_{B}\gg \sqrt{N_{0}s}$ corresponding to a low intensity bottleneck, we find the following approximation to the trajectory of the mutation burden immediately after the bottleneck re-expands.

$$\begin{array}{c} \langle x\left(t\right)\rangle ∼U_{d}\frac{\sqrt{4N_{0}}}{\sqrt{s}}(1-\frac{st}{2N_{B}})+U_{d}\frac{3st^{2}}{2N_{B}}+O\left(t^{3}\right) \end{array}$$ (15)

Concentrating on this second order expansion in *t*, the time after re-expansion from the bottleneck, we find that the curve first drops from its initial value $\langle x(0)\rangle =U_{d}\sqrt{\frac{4N_{0}}{s}}$, quickly reaches a minimum, and is then brought back up by the positive second order term. The location of the minimum is easily found to have the following parameter dependence.

$$\begin{array}{c} t_{min}\propto \sqrt{\frac{4N_{0}}{s}} \end{array}$$ (16)

The second derivative is positive at this extremum, implying a local minimum. Plugging *t*_*min*_ into our expression for 〈*x*(*t*)〉 in the limit *N*_0_ *s* ≫ 1, we find the following minimum value for the average number of recessive deleterious mutations per genome following a bottleneck.

$$\begin{array}{c} \langle x\left(t_{min}\right)\rangle ∼\theta_{0}(\frac{1}{\sqrt{4N_{0}s}}-\frac{1}{24N_{B}}) \end{array}$$ (17)

We note that ${\langle x\rangle }_{0}∼\frac{\theta_{0}}{\sqrt{4N_{0}s}}$ is the approximate mutation burden for the equilibrium distribution in the deterministic 2*N*_0_ *s* ≫ 1 limit, allowing us to simply write the extreme value of the *B*_*R*_ statistic as follows.

$$\begin{array}{c} {\begin{array}{c} B_{R}\left(t_{min}\right)∼\left(1-\frac{\sqrt{4N_{0}s}}{24N_{B}}\right) \end{array}}^{-1}\;>\;1 \end{array}$$ (18)

The burden ratio is again independent of mutation rate due to cancellation, as discussed above. We find the following dependence on time in immediate response to a population bottleneck.

$$\begin{array}{c} {B_{R}}^{recessive}\left(t\right)∼{\left(1-\frac{st}{2N_{B}}+\frac{3s^{3/2}t^{2}}{2N_{B}\sqrt{4N_{0}}}+O\left(t^{3}\right)\right)}^{-1}>1 \end{array}$$ (19)

This expansion is only valid in the small time limit where the quadratic term is subdominant, such that all values are positive. As seen in simulations described in the following section, for recessive deleterious mutations, the burden ratio remains positive at all times.

This precise result applies strictly in the limit of a strong, single-generation bottleneck, where *N*_0_ ≫ *N*_*B*_. Additionally, the technique used to compute integral expressions required the strong selection limit 2*N*_0_ *s* ≫ 1. Analysis of higher order contributions to the trajectory are made substantially easier by restricting to the low bottleneck intensity limit $2N_{B}>\sqrt{2N_{0}s}>1$, which may be biologically reasonable in human populations, for example, where many identified founding events are relatively short and on the order of *N*_0_ ∼ 10^4^ or *N*_*B*_ ∼ 10^3^, with the notable exception of the Out of Africa event (see further discussion in S1 Text on general dominance coefficients). Despite these analytic restrictions in parameter space, our simulations described below indicate that the signature of *B*_*R*_ > 1 is ubiquitous for populations under predominantly recessive selection.

#### Extended population bottlenecks

We argue that for the case of relatively low intensity bottlenecks, where intensity is defined as *I*_*B*_ ≡ *T*_*B*_/2*N*_*B*_ ≪ 1, we can approximately express the magnitude of *B*_*R*_ using a simple substitution (2*N*_*B*_)^−1^ → *I*_*B*_. This is equivalent to the claim that for low intensity bottlenecks, the *B*_*R*_ statistic depends only on the ratio of the bottleneck time to the bottleneck population size, and any explicit dependence on *T*_*B*_ occurs in subdominant contributions. This intuition is confirmed by simulations described in below, where we show that the accuracy of our analytic approximation breaks down as *I*_*B*_ → 1. For short bottlenecks with *I*_*B*_ < 1/10, the approximation of a single-generation sampling event remains sufficiently accurate, even for strong selective coefficients *s* ∼ 0.1. Under this trivially extended single-generation approximation, *B*_*R*_(*t*) can be written in terms of the intensity of a short bottleneck in the low intensity limit $I_{B}^{-1}>\sqrt{2N_{0}s}>1$ as follows.

$$\begin{array}{c} {B_{R}}^{extended}\left(t\right)∼{\left(1-I_{B}(st-\frac{3s^{3/2}t^{2}}{\sqrt{4N_{0}}}+O\left(t^{3}\right))\right)}^{-1}>1 \end{array}$$ (20)

The *B*_*R*_ of maximum effect, has a magnitude given approximately by,

$${\begin{array}{c} B_{R}{}^{extended}\left(t_{min}\right)∼\left(1-I_{B}\frac{\sqrt{N_{0}s}}{6}\right) \end{array}}^{-1}.$$ (21)

**Fig 2:**
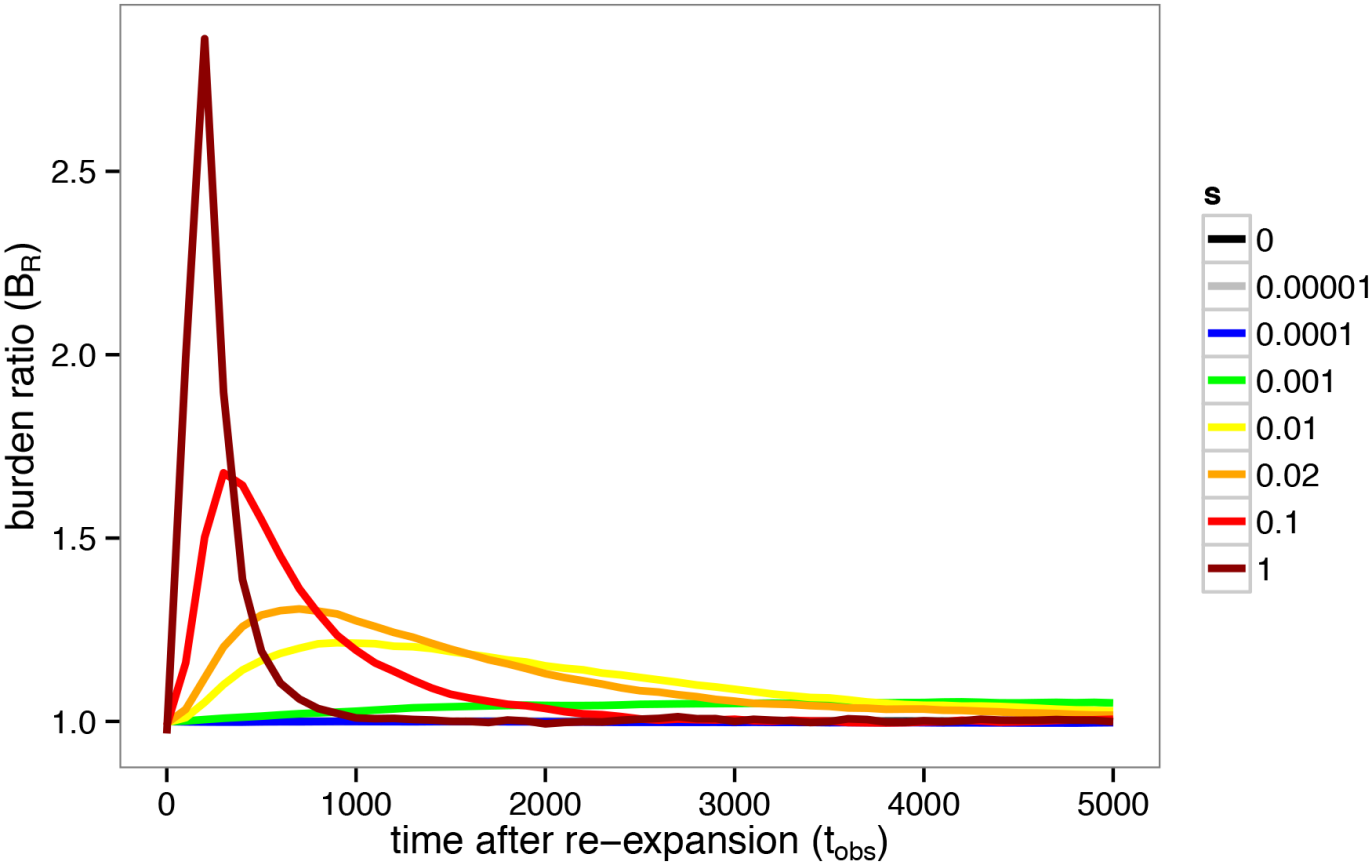
Time dependence of the *B*_*R*_ statistic after re-expansion. The time dependence of *B*_*R*_(*t*) after a population bottleneck is shown for for alleles under recessive selection (*h* = 0) for various selection strengths. Peak *B*_*R*_ values vary in both magnitude and time as a function of *s*. The founded population was simulated with 2*N*_0_ = 20000, 2*N*_*B*_ = 2000, and *T*_*B*_ = 200 and plotted for 5000 generations after re-expansion.

For illustration of the behavior described in the above analytics we present a time series of recessive simulations with curves representing various selection coefficients in Fig 2. The time dependence of the *B*_*R*_ statistic is plotted to demonstrate the simulated population’s response to a founder’s event. Crucially, we find that the peak *B*_*R*_ values vary in both magnitude and time as a function of *s*, as is consistent with our analytic understanding and intuition.

#### Transient response and time of observation determine detectable selection coefficients

Thus far, we have detailed the dynamic dependence of a set of alleles in a population, all with selective effect *s*, in response to demographic perturbation in the form of a bottleneck. Notably, for recessive selection, a peak response occurs in the *B*_*R*_ statistic at some time *t*_*min*_ after re-expansion. In general, both the magnitude of *B*_*R*_(*t*_*min*_) and the time of the peak itself depend sensitively on the selection coefficient. In general, a distribution of mutations with different selective effects will be present, many of which may be simultaneously polymorphic in a given population. Since alleles of different selective effect respond to the bottleneck on different time scales, one can ask what selective effect is most likely to be observed at a given time. For example, very strong selection has the tendency to peak and subsequently re-equilibrate immediately after the bottleneck, such that observation of alleles with large *s* is substantially more difficult at later times. On the other hand, alleles under relatively weak selection have a peak effect at very late times, such that at the time of data collection a statistically significant response may not yet have occurred.

**Fig 3:**
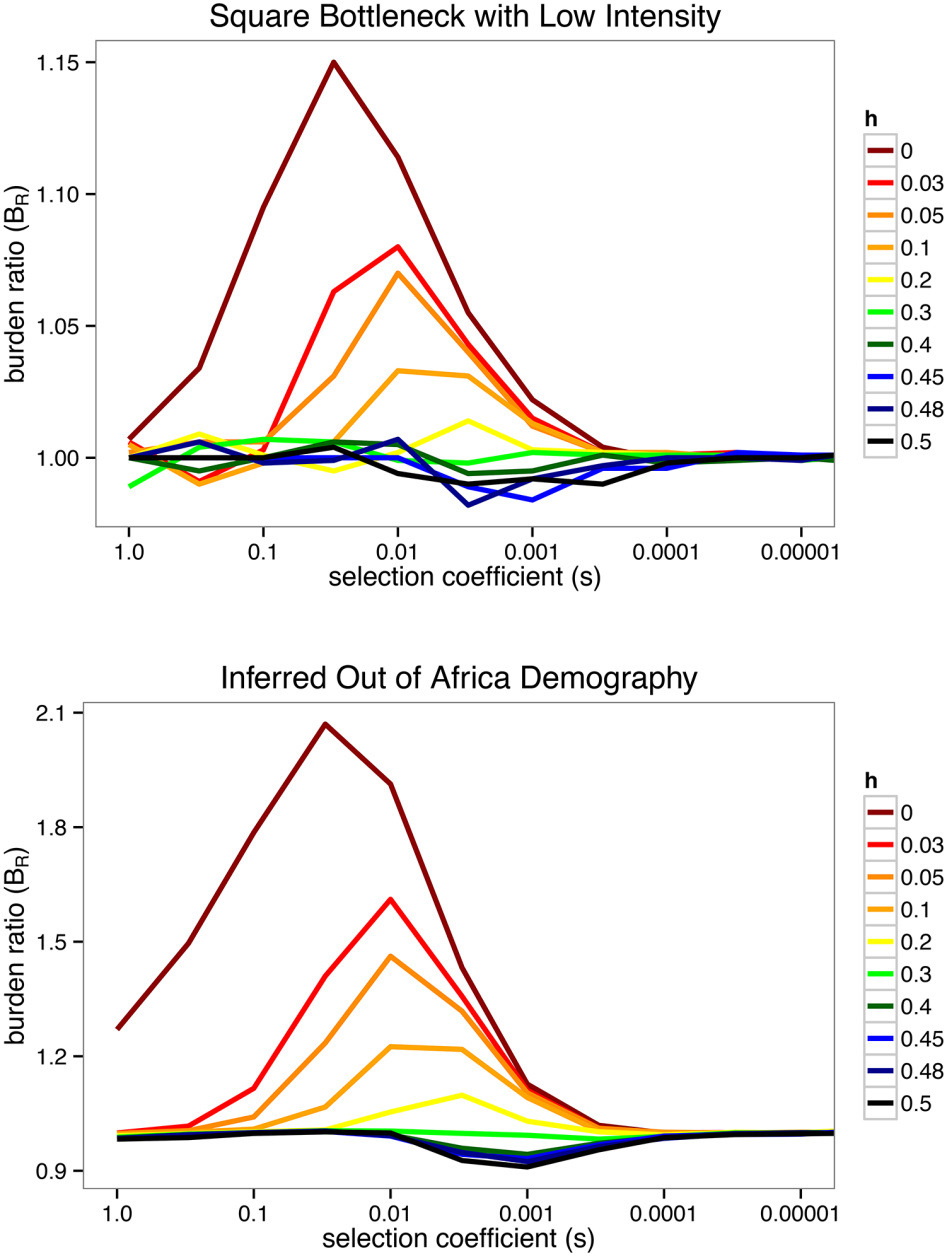
The *B*_*R*_ statistic at the time of observation. **ABOVE:** At the time of observation *t*_*obs*_, the value of *B*_*R*_(*t*_*obs*_) is plotted as a function of the average strength of selection *s* and dominance coefficient *h*. Dominance coefficients appear as solid lines with fully recessive selection (*h* = 0) at the top and purely additive selection ($h=\frac{1}{2}$) at the bottom. For strong selection *B*_*R*_ → 1 due to the rapid transient response. For weak selection *B*_*R*_ → 1 due to the nearly neutral insensitivity to the bottleneck. For some intermediate dominance coefficient *h*_*c*_, a critical value occurs (*h*_*c*_ ∼ 0.25 in the example shown, but explored more generally in S1 Text) where additive and recessive effects cancel, yielding *B*_*R*_(*h*_*c*_) ∼ 1. A low intensity bottleneck (*I*_*B*_ = 0.05) is shown, with parameters 2*N*_0_ = 20000, 2*N*_*B*_ = 2000, *T*_*B*_ = 100, and *t*_*obs*_ = 1000. **BELOW:** The same range of parameters is plotted for a realistic demographic model of the Out of Africa event comparing Africans and Europeans [48], where *B*_*R*_ = 〈*x*〉_*African*_/〈*x*〉_*European*_. The European bottleneck has estimated intensity *I*_*B*_ ∼ 𝒪(0.5), an order of magnitude stronger than the simple bottleneck above, allowing for potentially observable deviations from *B*_*R*_ ∼ 1 if a large fraction of analyzed variants act recessively with *h* < *h*_*c*_ ∼ 0.25.

We would like to understand the transient behavior of the burden ratio *B*_*R*_(*t*), as well as the value of the selection coefficient *s* for which *B*_*R*_ is largest at a given time. When comparing theory to population data, one has little control over the demographic history, and thus it becomes important to understand the selective coefficient that dominates at the time of observation. According to the time dependent expression in Eq (20), we expect the effect to decrease quite rapidly for very large *s*. However, the peak occurs quite early in the case of larger *s* values, allowing the mutation burden to equilibrate over a longer period of time between the peak and observation to return to mutation burden values close to *B*_*R*_ ∼ 1. This tells us that the equilibration process is what reduces the magnitude of *B*_*R*_ for large *s*. In the case of very recent bottlenecks, the large *s* values dominate, but for later times of observation, this signal has partially equilibrated, potentially allowing a smaller *s* value to dominate the statistic. At a given time of observation *t*_*obs*_, one can represent *B*_*R*_(*s*, *t*_*obs*_) as a function of various selection coefficients *s*. Fig 3 represents *B*_*R*_(*s*) for a fixed *t*_*obs*_ for various dominance coefficients *h*. We concentrate here on recessive variation with *h* = 0, but note that a critical value occurs at some *h*_*c*_ where additive and recessive effects offset each other in the *B*_*R*_ statistic, the dynamics of which are detailed in S1 Text and illustrated in S1 Fig). Based on our analytics, we expect the peak to shift from extreme high *s* values at early times to extreme low *s* values at late times, eventually dissolving into neutrality. We take the *s* derivative of Eq (20) to find the maximum at *t*_*obs*_.

$$\begin{array}{c} \partial_{s}B_{R}\left(s,t_{obs}\right){|}_{s=s_{max}}\propto -I_{B}t_{obs}+\frac{9s^{1/2}I_{B}t_{obs}^{2}}{2\sqrt{4N_{0}}}=0 \end{array}$$ (22)

$$\begin{array}{c} s_{max}∼\frac{16N_{0}}{81t_{obs}^{2}}∼\frac{2N_{0}}{10t_{obs}^{2}} \end{array}$$ (23)

One can easily show that the second derivative evaluated at this point is negative, confirming that this is a maximum. This result matches our intuition: maximum *s* values of *B*_*R*_(*s*, *t*) are found at high *s* for early times, *s*_*max*_(*t* → 0) ≫ 1, and at low *s* for late times, *s*_*max*_(*t* → ∞) ≪ 1. This is qualitatively observed in our simulations by comparing the relative values of *B*_*R*_(*s*) as a function of time.

As the effect is transient, we can define a relaxation time *t*_*relax*_ corresponding to the vanishing of any response to the bottleneck. This is given by determining when *s*_*max*_ is dominated by effectively neutral variation at roughly *s*_*max*_ ∼ 1/2*N*_0_. After this time, *B*_*R*_(*s*, *t*) cannot be differentiated from one for any *s*.

$$\begin{array}{c} t_{relax}<\frac{2N_{0}}{\sqrt{10}}<2N_{0} \end{array}$$ (24)

We note that the return to equilibrium happens on a time scale faster than random drift, even for the weakest selective effects, thus validating our perturbative approximations using *t*/2*N*_0_ ≪ 1. Higher order time dependence in Eq (20) may substantially correct this estimate, but we feel that the presentation of this methodology is conceptually important and provides a greater understanding of the transient dynamics of population response to bottlenecks. As it is relevant to human populations, we note that if both populations expand exponentially after the bottleneck, the effect may persist long beyond *t*_*relax*_. This is explored analytically in the S1 Text.

### Comparison of analytic results to simulations

**Fig 4:**
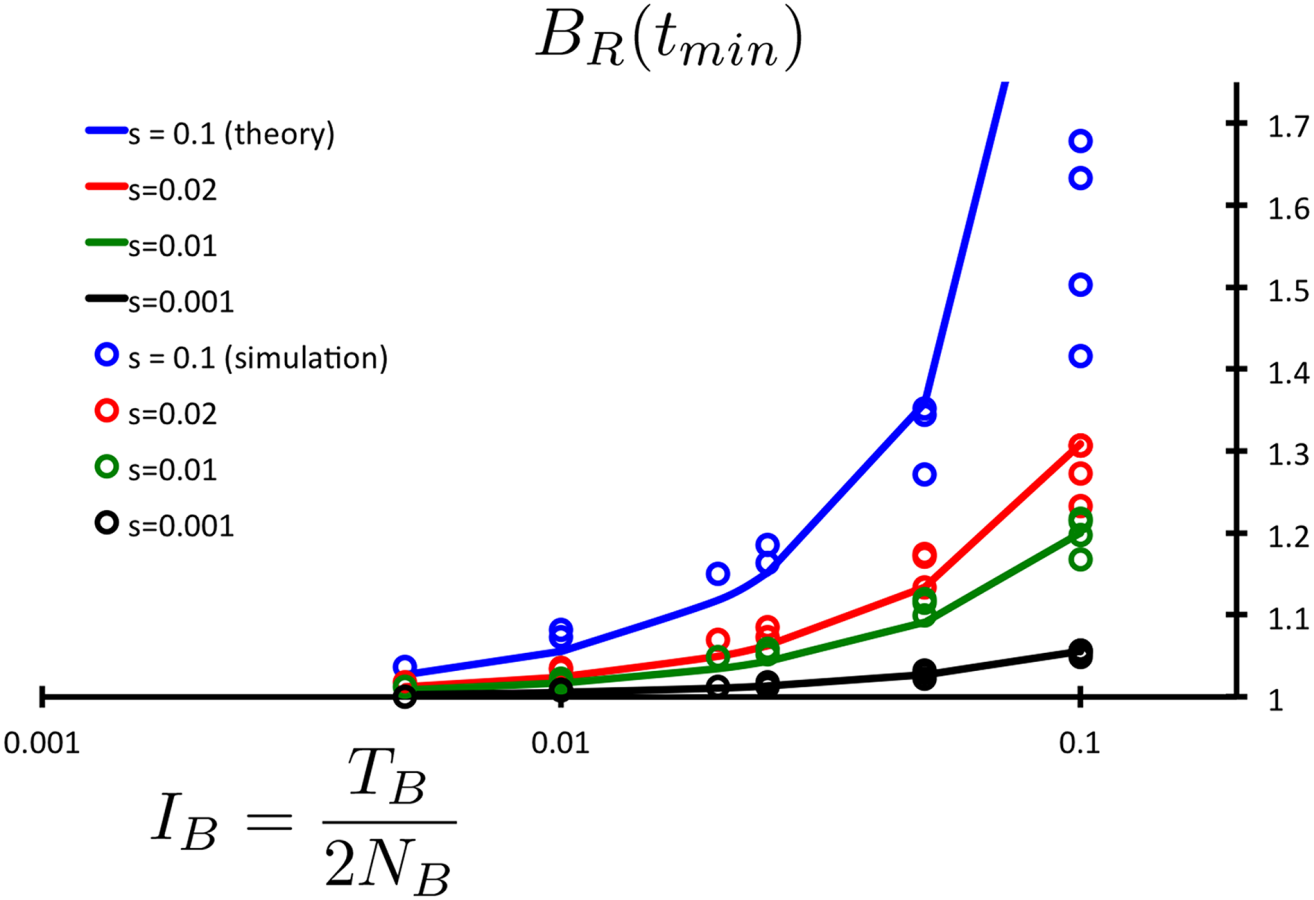
Comparisons of analytic and simulation results. Maximum response values of the burden ratio *B*_*R*_(*t*_*min*_) are plotted for recessive selection as a function of bottleneck intensity. A wide range of parameter sets is plotted with all combinations of 2*N*_*B*_ = {2000,1000,400,200,100}, *s* = {0.1,0.02,0.01,0.001}, *T*_*B*_ = {200,100,50,20,10}, each simulated for 10^8^ nucleotide sites. For relatively low intensity bottlenecks we note excellent agreement over the parameter ranges plotted. Intensities with *I*_*B*_ = *T*_*B*_/2*N*_*B*_ > 0.1 are excluded, as the single-generation bottleneck scaling breaks down in favor of a long bottleneck scaling. The approximation necessarily weakens for simulations that represent longer bottlenecks, and only for strong selective coefficients, as expected. This quantifies the limitations of the single-generation bottleneck approximation, as we observe substantial deviation only around *I*_*B*_ = 0.1 and with selection strength *s* = 0.1.

We checked our analytic results using a forward time population simulator, described in detail in the S1 Text. Given the ubiquity and analytic simplicity of the exponential decay in the additive scenario, we focus here on our predictions for recessive variation. We compare analytic expressions of *B*_*R*_(*t*_*min*_) at the peak response given in Eq (21) for various selection coefficients. We simulated a wide range of bottleneck parameters to probe the limitations of our theoretical understanding. In Fig 4, we demonstrate the accuracy of our analytic results, by plotting the ratio of the simulated values of *B*_*R*_(*t*_*max*_, *s*, *I*_*B*_) to our analytic predictions *B*_*R*_(*t*_*max*_, *s*, *I*_*B*_) as presented in Eq (21). We arrange our simulated data by bottleneck intensity *I*_*B*_, as we expect the single-generation bottleneck approximation to break down as intensity is increased due to longer bottleneck duration *T*_*B*_ ≫ 1. As plotted, complete agreement between simulated data and analytic predictions is represented by a flat line at ${B_{R}}^{sim}/{B_{R}}^{analytic}=1$. As expected, we find deviations as we approach the limitations of our perturbative approximation, roughly around *T*_*b*_ ∼ 2*N*_*B*_/10 when *I*_*B*_ ∼ 0.1. Below these higher intensities, we find quite good agreement for all parameter sets well below 10% error, even at *I*_*B*_ = 0.05. Further comparison between simulation and analytic results is presented in S1 Text and illustrated in S2 Fig.

### Empirical detection of recessive selection

The *B*_*R*_ statistic provides a qualitative indication of recessive selection (*h* ≪ 0.5), in that values over one theoretically correspond to recessivity. This corresponds to a reduction in the average number of deleterious alleles per haploid locus in a founder population relative to a non-bottlenecked population. To test whether the statistic is sensitive to recessive selection, we analyze human exome data from the Exome Sequencing Project (ESP) [37]. We compare European Americans (EA), known to have undergone a relatively intense bottleneck during the “Out of Africa” event, to African Americans (AA), who have substantial African ancestry that did not experience this founder’s event. We aggregate a set of genes and compute the per-haploid mutation burdens, 〈*x*〉_*AA*_ and 〈*x*〉_*EA*_ for each gene set by summing the frequencies of all variants occurring in those genes within the AA and EA populations separately, such that ${\langle x\rangle }_{AA}\equiv \sum_{i}x_{i}^{AA}$ and ${\langle x\rangle }_{EA}\equiv \sum_{i}x_{i}^{EA}$. This provides a group burden ratio score $B_{R}\equiv \sum_{i}x_{i}^{AA}/\sum_{i}x_{i}^{EA}$ for the entire gene set ranging from predicted additive (or dominant) with *B*_*R*_ < 1 to predicted recessive with *B*_*R*_ > 1. While this strategy could in principle be applied directly to a single gene, substantial statistical fluctuations tend to make this measure unreliable on the individual gene level.

We assemble sets of genes associated with known autosomal recessive (AR) diseases, some of which are potentially under recessive selection, and compute a corresponding *B*_*R*_ score. In the absence of pleiotropy and the presence of purifying selection against these disease phenotypes, we naively expect these genes to act under partial (*h* < 0.5) or total recessive selection (*h* ≈ 0). We check for significant deviation from *B*_*R*_ = 1 in several gene sets: 44 genes associated with diseases with “autosomal recessive” in the name of the disease with at least 5 annotated variants in the Human Gene Mutation Database (HGMD), 37 genes associated with congenital hearing loss (HL) and found only with AR mode of inheritance in a clinical genetics lab, and 1348 genes with Clinical Genomic Database (CGD) AR annotations [38–40]. Additionally, we aggregate non-overlapping HGMD and HL genes into a larger combined list of 72 genes.

To compute *B*_*R*_ gene scores, we assume that derived variants at a given locus are deleterious, and include derived alleles of all frequencies, including those fixed in one or both of the populations. We restrict our analysis to nonsense variants and non-synonymous variants predicted to be damaging using a human-free version of PolyPhen2 [36] developed to remove bias due the ancestry of the human reference. Derived alleles fixed in one of the two populations are included in the analysis of the burden, as they contribute to the weighted mean 〈*x*〉.

We estimate significance using bootstrapped standard errors, as described in detail in the S2 Text. First, we compute the burden ratio for all genes in the genome, and find no statistical deviation from one, replicating previously published results [35, 36]. Analysis of the CGD gene set again shows no statistically significant deviation from one. Given the whole genome result, this is not unexpected, as this set of over 1000 genes is plausibly large enough to representatively sample the set of all genes. It is likely that many of these genes have only one or a few variants under recessive selection, with the rest being neutral or even dominantly acting. In contrast, we find statistically significant *B*_*R*_ > 1 values in the potentially more reliable HGMD and HL gene sets, despite their small size, as well as in the combined set. We partially replicate our results from ESP using an independent dataset, from the 1000 Genomes Project (1KG), again finding statistical significance in the HGMD disease gene set [41]. A detailed discussion of the data sets and statistical analyses used is provided in S2 Text and detailed in S1 Table (with full gene lists included in a supplemental spreadsheet).

We find statistical significance for two separately obtained disease gene sets, as well as in the combined set. The HGMD gene set is significant in both ESP and 1KG. Additionally, we find null results in nearly all controls presented in S2 Text and detailed in S2 Table. Together, the empirical analysis provides suggestive evidence that genes associated with autosomal recessive disease and thus potentially under recessive selection can show significant burden ratio values *B*_*R*_ > 1. The resulting analysis is summarized in Table 1. In light of these findings, we believe we have demonstrated the potential usefulness of this method for identifying sets of genes under recessive selection.

**Table 1:** Empirical results for autosomal recessive disease gene sets.

PolyPhen damaging and stop variants only			
**ESP**	Number of genes	*B* *_R_* score	Bootstrap p-value
All Genes	14722	1.010	0.105
AR Clinical Genes from CGD	1205	1.005	0.440
HGMD (>5 var) “Autosomal Recessive”	37	**1.215** * ^†^	**0.018** * ^†^
AR Hearing Loss from LMM	30	**1.190** *	**0.003** *
Combined HGMD and Hearing Loss	60	**1.168** *	**0.006** *
**1KG**	Number of genes	*B* *_R_* score	Bootstrap p-value
All Genes	16985	0.993	0.756
AR Clinical Genes from CGD	1348	0.960	0.833
HGMD (>5 var) “Autosomal Recessive”	44	**1.236** * ^†^	**0.047** * ^†^
AR Hearing Loss from LMM	37	1.004	0.471
Combined HGMD and Hearing Loss	72	1.135	0.111

* **significant**

^†^ **replicated**

Tabulated results are presented for the *B*_*R*_ statistic applied to several gene sets associated with autosomal recessive diseases. We restrict to damaging and nonsense variants and use a bootstrap p-value to determine significance. We find statistically significant results in two data sets and a set combining the two, suggesting the potential for detection of recessive selection under the assumption that many known recessive human diseases are under recessive purifying selection. Details are provided in S2 Text.

Given the significant observed values of *B*_*R*_ > 1 in these gene sets, one can gauge the degree of recessivity for a given set. Specifically, we can readily estimate the average dominance coefficient for damaging and nonsense mutations within a set of genes under the assumption that these mutations all act with a single average dominance coefficient $\bar{h}$ and an average selection strength $\bar{s}$. We caution that estimates using a single *h* and *s* pair of values for all derived mutations may be inappropriate if there is substantial variance in either or both of these parameters. In the absence of information about the variance in dominance coefficients, we believe this approximation may still be informative (if only as a rough guide) in gene sets that clearly deviate from neutrality. Given the details of the Out of Africa demography, the data for the HGMD gene set are consistent with an average dominance coefficient ${\bar{h}}_{HGMD}≲0.2$ (with 95% confidence), however, this bound is conservative over all possible values of the average strength of selection in this gene set. For average selective strengths of ${\bar{s}}_{HGMD}=\{0.001,0.01,0.1\}$ in damaging and nonsense variants, we find that the corresponding allowed average dominance coefficients are ${\bar{h}}_{HGMD}≲\{0.15,0.2,0.05\}$ (with 95% confidence), respectively. Note that the non-monotonicity in these values is a consequence of the behavior shown for the Out of Africa demography in Fig 3. Additionally, all average dominance coefficients for HGMD are inconsistent with weak average selective strengths below roughly ${\bar{s}}_{HGMD}∼0.0003$. Complementary population data from distinct founder’s events may provide stricter bounds on both the average dominance coefficients and average selective strengths for a given gene set.

## Discussion

The increase in prevalence of recessive phenotypes following population bottlenecks has attracted the interest of geneticists for a long time [19, 42]. Theoretical analysis of allele frequency dynamics in a population expanding after a bottleneck suggested that frequency of an individual allele may rise due to increased drift [42–44]. Here, we focus on a more general question of the collective dynamics of recessively acting genetic variation. In line with the qualitative description found in [31], our analysis suggests that the number of recessively acting variants per haploid genome is reduced in response to a bottleneck and subsequent re-expansion. Generally, we have demonstrated that features of the derived allele spectrum of recessive deleterious polymorphisms behave distinctly from additively acting variation following a population bottleneck and subsequent re-expansion. The response of additive variation depends crucially on the average number of deleterious alleles, and on the number of generations for which selection is relaxed during the bottleneck. In contrast, the dynamics of recessive variation crucially depend on the variance of the site frequency spectrum, rather than the average number of mutations per individual, such that the accumulation of deleterious mutations can respond strongly even to a single-generation bottleneck. Importantly, the temporal dynamics of the accumulation of deleterious alleles depends qualitatively on dominance coefficient and quantitatively on selection coefficient. The qualitative dependence on dominance coefficient suggests that one can learn about recessivity from analysis of the population dynamics in response to a founder‘s event. If the variation is additive, the number of deleterious variants per a haploid genome is larger in a bottlenecked population than in a corresponding equilibrium population. If the variation acts recessively, this number is smaller. The selection coefficient determines the timing of response to a bottleneck.

By explicitly analyzing the non-equilibrium response to a bottleneck, we suggest that naively confounding demographic features may actually shed light on underlying population genetic forces. In realistic populations, for example in modern humans, substantial work has been done to identify and understand the recent demographic history of geographically disparate populations [37, 45–54]. In a recent paper, Simons, et al. [35] use the *B*_*R*_ statistic on the whole genome level to empirically compare the accumulation of mutations in European Americans and African Americans. The authors find no statistically significant differences in the whole genome mutation burden of these populations, a result that was extended to all two-point comparisons between a diverse set of humans by Do, et al. [36]. To explain this observation, Simons, et al. derive a complementary theoretical treatment of the dynamics of segregating alleles using branching process techniques and extensive simulations, providing results that are consistent with those presented here.

In the case of the “Out of Africa” event, a historically substantiated and believable demographic model can be used to understand the difference between African and European populations since their divergence. The comparison between populations that have and have not undergone a bottleneck can be used to elucidate plausible selection and dominance coefficients by making use of a simulated version of this demography. As shown in Fig 3 for the comparison between Africans and Europeans, a realistic demographic model can be used to bound the selection and dominance coefficients in modern populations based on a single observation, such as those detailed in [35, 36].

Although the net number of recessive deleterious mutations is reduced as a consequence of a founder‘s event and subsequent re-expansion, the fitness of individuals carrying these alleles is not necessarily increased, as the number of homozygotes is known to increase after a population bottleneck. However, the number of heterozygous deleterious sites, or the average carrier frequency for associated alleles, is suppressed, such that the mating of individuals from disparate bottlenecked populations may result in a decreased incidence of recessive phenotypes in such mixed lineages. In studies of model organisms, this may have applications when comparing laboratory populations founded from a few wild type individuals to their corresponding natural populations.

We have demonstrated that analysis of the *B*_*R*_ statistic on the gene set level shows significant deviations above one in genes known to be responsible for autosomal recessive human disease. In principle, the results of this study can be extended to the analysis of any specific groups of genes beyond those with a known mode of inheritance. Sufficiently large subsets of alleles that are medically relevant may be analyzed in humans to identify the mode of selection for candidate variants of potentially recessive diseases.

In sum, the non-equilibrium dynamics induced by demographic events is an essential, and indeed insightful, feature of most realistic populations. Population bottlenecks, abundant in laboratory populations and in natural species, have the potential to provide a novel perspective on the role of dominance in genetic variation.

## Methods

**Simulation details.** We performed analysis using a forward time population simulator, custom written in **C**, available at http://genetics.bwh.harvard.edu/wiki/sunyaevlab/dbalick. For computational speed, the simulator only keeps track of allele frequencies in a freely recombining diploid system, rather than containing full genome information. We use an infinite sites model with a mutation rate of 2 × 10^−8^ per generation per site. Allele counts in the current generation are sampled based on the frequencies in the previous generation *x*_*old*_, the selection coefficient *s*, and the dominance coefficient *h*. We calculate the expected frequency *x*_*current*_ in the current generation as:

$$\begin{array}{c} x_{current}=\frac{\left(x_{old}^{2}\left(1+s\right)+x_{old}\left(1-x_{old}\right)\left(1+s\right)h\right)}{\left(x_{old}^{2}\left(1+s\right)+2x_{old}\left(1-x_{old}\right)\left(1+s\right)h+{\left(1-x_{old}\right)}^{2}\right)}. \end{array}$$ (25)

The simulator has arguments for per base mutation rate *U*_*d*_, selection coefficient *s*, and dominance coefficient *h*, with a default burn-in of 300,000 generations where sampling occurs every 100 generations in sped-up mode before transitioning to sampling every 1 generation at 1000 generations before time *t* = 0.

The code was designed to allow for flexible demographic histories, in order to accurately represent events such as the “Out of Africa” migratory event in human population genetic history. For the purposes of comparison to our analytic results, we ran simulations for a simple, square bottleneck of varying population sizes for both the equilibrium population with size 2*N*_0_ = 2 × 10^4^ and bottlenecked populations with temporarily reduced sizes of 2*N*_*B*_ = {2000,1000,400,200,100} for a duration of *T*_*B*_ = {200,100,50,20,10} generations. These simulations were performed under both purely additive (*h* = 0.5) and purely recessive (*h* = 0) selection, for a wide range of selection coefficients *s* = {1,0.1,0.02,0.01,0.001}. For simulations of a range of selective effects and dominance coefficients shown in Fig 3, we used a square bottleneck with parameter 2*N*_0_ = 20000, 2*N*_*B*_ = 2000, *T*_*B*_ = 100, and *t*_*obs*_ = 1000 and a realistic Out of Africa demography detailed in Tennessen, et al. [48].

**Human polymorphism data.** We analyze exome data from the Exome Sequencing Project (ESP) and validate some of our findings using exome data from the 1000 Genomes Project (1KG)[37, 41]. We use available frequency information for polymorphic variants to compute an average per haploid mutation burden per gene for all genes in ESP in 1088 European Americans(EA) with largely European ancestry and 1351 African Americans (AA) with substantial African ancestry. In 1KG, we compare 85 Northern Europeans from Utah (CEU) to 88 Yorubans (YRI) by computing the same statistic. We sum these mutation burdens over genes of interest to compute an aggregate *B*_*R*_ score for a given gene set.

**Human-free Polyphen2.** To compute mutation burden gene scores for putatively deleterious mutations, we restrict our analysis to non-synonymous nonsense variants and variants predicted to be damaging using a human-free version of PolyPhen2 [36]. This software was developed to remove bias due to the mixed ancestry of the human reference sequence, and annotates derived alleles based on chimpanzee orthologs.

**Disease gene sets.** We use several lists of genes associated with AR diseases that we naively expect to act under partial or total recessive selection. First we compile a set of genes from the Human Gene Mutation Database (HGMD) only associated with diseases with “autosomal recessive” in the disease name [38]. We restrict this set to genes with at least 5 disease-associated variants to guarantee sufficient polymorphism and reduce noise in the *B*_*R*_ statistic. This set contains 38 genes that appear in the list of ESP scored genes (44 in 1KG) and is referred to as “HGMD”. We use Congenital Hearing Loss as an example of a polygenic, largely recessive disease. We obtained an annotated gene list of AR genes associated with hearing loss from the Laboratory for Molecular Medicine (LMM) [39]. This list contains 30 genes in ESP (37 in 1KG) and is referred to as “Hearing Loss”. Notably, this list excludes connexin 26 (GJB2), among other genes, which has additional association with AD hearing loss. Additionally, we assemble a combined list of all genes from HGMD and Hearing Loss, with a total of 60 genes in ESP (72 in 1KG) after removing overlap, referred to as “Combined”. To assemble a larger, though noisier gene set, we use all annotated AR genes in the Clinical Genomic Database, referred to as “CGD”, which contains 1268 genes in ESP and 1348 genes in 1KG [40].

## Supporting Information

S1 Text Analytic and simulation details. Additional analytic details are provided here. A discussion of the dynamics of general moments of the site frequency spectrum is included, followed by a detailed calculation of the time dependent trajectory of the mutation burden and burden ratio in the case of recessive selection. Generalizations to distributions of selective effects and dominance coefficients are included. The case of a long bottleneck is described, in addition to a discussion of the effect of exponential expansion on the mutation burden and burden ration. Relevant Gaussian integrals are listed. We detail the curve collapse comparison of analytic results to simulations. For the reader’s convenience, a list of relevant variables is included.
(PDF)

S2 Text Data analysis details. Here we describe further details of the analyzed data. Gene sets are discussed in detail, and are included in a supplemental file. We discuss the results for *B*_*R*_ per gene set (with corresponding standard errors), and include an analysis of synonymous sites as a negative control for damaging and nonsense sites in these gene sets.
(PDF)

S3 Text Simulation code. Here we present the simulation code for convenience. This can also be found at http://genetics.bwh.harvard.edu/wiki/sunyaevlab/dbalick.
(PDF)

S1 Data Gene set lists for human disease data. Here we present lists of genes used in our analysis: A list of all genes annotated with human-free PolyPhen2 [36], all genes annotated as Autosomal Recessive (AR) in the Clinical Genomics Database (CGD) [40], genes that appear in the Human Gene Mutation Database (HGMD) with diseases with “autosomal recessive” in the name [38], hearing loss genes annotated as AR by the Laboratory for Molecular Medicine (LMM) [39], and a combined list of HGMD and hearing loss genes.
(XLSX)

S1 Fig Dynamics of the critical dominance coefficient *h*_*c*_. **ABOVE:** *B*_*R*_ is plotted for several values of dominance coefficient *h* as a function of time after re-expansion from the bottleneck to demonstrate the observable range on either side of the critical dominance coefficient. Additive and recessive alleles are distinguishable when observing at early times prior to re-equilibration due to additive selection. During the equilibration process, the critical value of the dominance coefficient *h*_*c*_(*t*) at which *B*_*R*_ = 1 shifts from near pure recessivity (*h*_*c*_ ∼ 0) at early times to near additivity at late times (*h*_*c*_ = 1/2). After additive re-equilibration, partially recessive alleles are still detectable (*B*_*R*_ > 1) with purely recessive alleles providing the largest signature prior to their eventual equilibration. In this plot 2*N*_0_ = 20000, *s* = 10^−2^, *T*_*B*_ = 100 and 2*N*_*B*_ = 2000 such that *I*_*B*_ = 0.05. This qualitative behavior is generic for most parameter values in the short, low intensity bottleneck limit *I*_*B*_ ≪ 1, however the time dependence of *h*_*c*_ depends sensitively on these parameters.**BELOW:** The critical dominance coefficient *h*_*c*_ is plotted as a function of time. At early times *h*_*c*_ ∼ 0 is close to pure recessivity. After re-equilibration of additive alleles, *h*_*c*_ ∼ 1/2, such that only partially recessive alleles provide a signature. Any value *B*_*R*_ > 1 provides evidence of alleles under partially recessive selection, with the largest contribution coming from purely recessive alleles.
(TIFF)

S2 FigCurve collapse for *B*_*R*_(*t*_*min*_).
**ABOVE:** Here we plot a curve collapse for the peak response *B*_*R*_(*t*_*min*_) to compare our analytic description to simulated data. Values near ${B_{R}}^{sim}/{B_{R}}^{analytic}=1$ validate our analytic description. Deviation from this line represents a breakdown in the proposed scaling as a function of the intensity and selective effect. We find that the collapse is weakly stratified by selective coefficient, even in the range of good agreement at low intensity. Large selective coefficients *s* = 0.1 deviate fastest, implying a breakdown in the short bottleneck scaling of *B*_*R*_(*s*). Parameter values of 2*N*_*B*_ = 2000, *T*_*B*_ = {200,100,50,20}, and *s* = {0.1,0.02,0.01,0.001} are included on the plot. *B*_*R*_(*t*_*min*_(*s*)) occurs at different times *t*_*min*_(*s*) for different selection coefficients.**BELOW:** ${B_{R}}^{min}$ curve collapse is plotted as a function of rescaled intensity $\sqrt{s}I_{B}$ to illustrate that breakdown of our theoretical predictions occurs in the limit $I_{B}\gg 1/\sqrt{2N_{0}s}$, where *N*_0_ is fixed in this collapse for illustrative purposes.(TIFF)

S1 Table
${B_{R}}^{dam}$ data table for damaging and nonsense sites.Here we apply the *B*_*R*_ statistic to sets consisting of genes known to be associated with autosomal recessive (AR) disease, as well as to a set of all genes in the genome. Only nonsense and human-free PolyPhen2 damaging variants are counted in these African and European population samples. Some results acquired from ESP data are replicated in 1KG, despite smaller population samples. For comparison, we display results of the paired Student t-test, which shows weaker ability to distinguish between distinct average mutation burdens in comparisons between African and European samples.(TIF)

S2 Table
${B_{R}}^{synon}$ data table for fourfold degenerate synonymous sites.All analyses are repeated for a *B*_*R*_ statistic computed using only fourfold degenerate synonymous variants assumed to be under little or no selection. 1KG shows slight significance when testing all genes, however the value of *B*_*R*_ remains very close to one, potentially indicating spurious significance. Naively, this provides a control for the results derived from nonsense and damaging variants above in the absence of selection or linkage to selected sites.(TIF)
