# Supplementary material for: Dominance of Deleterious Alleles Controls the Response to a Population Bottleneck
Source: PLoS Genet. 2015 Aug 28;11(8):e1005436. doi: 10.1371/journal.pgen.1005436 (PMC4552954; doi:10.1371/journal.pgen.1005436)
Supplement: S1 Text — Additional analytic details are provided here. A discussion of the dynamics of general moments of the site frequency spectrum is included, followed by a detailed calculation of the time dependent trajectory of the mutation burden and burden ratio in the case of recessive selection. Generalizations to distributions of selective effects and dominance coefficients are included. The case of a long bottleneck is described, in addition to a discussion of the effect of exponential expansion on the mutation burden and burden ration. Relevant Gaussian integrals are listed. We detail the curve collapse comparison of analytic results to simulations. For the reader’s convenience, a list of relevant variables is included. (PDF) [file pgen.1005436.s001.pdf]

Text S1. Analytic and simulation details.

Integration of the Kolmogorov Equation

The Forward Kolmogorov Equation is generally used to describe the probability distribution of the trajectory of an allele in frequency space within a population [33,55]. If one analyzes the frequency of all polymorphic alleles in the population, or the site frequency spectrum (SFS), one can describe the collective dynamics of this distribution in a very similar form using an infinite sites model. The Kolmogorov equation describes the time dependence of the probability density $\rho(x,t)$ of an allele's frequency x at some time t. In the limit of a large number of simultaneously polymorphic alleles, one can think of all points in this probability distribution as being filled by one or more alleles. Choosing to focus on the case of purely recessive variation, one can write down the time dependence of the SFS in the following form.

$$\partial_t \phi(t,x) = s\partial_x \left(x^2(1-x)\phi\right) + \frac{1}{4N}\partial_x^2(x(1-x)\phi) + 2NU_d\,\delta\left[x - \frac{1}{2N}\right] \tag{26}$$

The presence of the delta function represents an influx of new mutations into the spectrum at initial frequency $1/2N$ coming from $2N$ individuals in the population, each with a mutation rate U_d per individual per generation. This acts like a source in the SFS at $x = 1/2N$, and is a reasonable approximation in the limit of a long genome with no double mutations or back mutations. We are interested in the time dependence of specific moments of this distribution. For example, to determine the time dependence of the first moment of the distribution $\langle x \rangle$, we multiply by x and integrate to find the time dependence of this moment.

$$\partial_t \langle x \rangle = \int dx\, x\, s\partial_x \left(x^2(1-x)\phi\right) + \int dx\, x\, \frac{1}{4N}\partial_x^2(x(1-x)\phi) + \int dx\, x\, 2N_0 U_d\,\delta\left[x - \frac{1}{2N_0}\right] \tag{27}$$

The delta function integral is trivially computed and we integrate by parts once on each of the other integrals, noting that the boundary term at $x = 0$ vanishes due to the $x^2(1-x)\phi(x)$ factor under the derivatives and the rapid decay of $\phi(x)$ ensures approximate vanishing of the boundary term at $x = 1$, which scales as x at low frequencies $x \to 0$ and decays rapidly as $x \to 1$ provided selection is efficient.

$$\partial_t \langle x \rangle = -\int dx\, s\left(x^2(1-x)\phi\right) - \int dx \frac{1}{4N}\partial_x(x(1-x)\phi) + U_d \tag{28}$$

The drift term can be identified as a total derivative, which vanishes, leaving the following dynamical equation for the mutation burden.

$$\partial_t \langle x(t) \rangle^{recessive} = U_d - s\left(\langle x(t)^2 \rangle - \langle x(t)^3 \rangle\right) \tag{29}$$

The time dependence of all higher moments can be computed in a completely analogous way. Since it is relevant for our present purposes, we note the equation of motion for the second non-central moment.

$$\partial_t \langle x^2(t) \rangle^{recessive} = \frac{U_d}{2N} - 2s\left(\langle x(t)^3 \rangle - \langle x(t)^4 \rangle\right) + \frac{1}{2N}\left(\langle x(t) \rangle - \langle x^2(t) \rangle\right) \tag{30}$$

Equations of motion for moments of the site frequency spectrum of alleles under purely additive selection can be computed in the same way. Here we cite these results for convenience. Note that we are using the convention $s_{add} \equiv hs = s/2$

$$\partial_t \langle x(t) \rangle^{additive} \approx U_d - \frac{s}{2}\left(\langle x(t) \rangle - \langle x^2(t) \rangle\right) \tag{31}$$

$$\partial_t \langle x^2(t) \rangle^{additive} \approx \frac{U_d}{2N} - s\left(\langle x^2(t) \rangle - \langle x^3(t) \rangle\right) + \frac{1}{2N}\left(\langle x(t) \rangle - \langle x^2(t) \rangle\right) \tag{32}$$

In the limit $\sqrt{2Ns} \gg 1$, the SFS for recessive alleles rapidly vanishes at high frequencies such that we can drop the $(1-x)$ dependence to find the following approximate equation of motion.

$$\partial_t \langle x(t) \rangle^{recessive}_{\sqrt{2Ns} \gg 1} \approx U_d - s\langle x(t)^2 \rangle \quad \text{for } \sqrt{2Ns} \gg 1 \tag{33}$$

The $(1-x)$ contribution in the dynamics of higher moments can be similarly neglected. For alleles under additive selection, the analogous strong selection limit is given by$2Ns \gg 1$, which results in the following simplified dynamics.

$$\partial_t \langle x(t) \rangle^{additive}_{2Ns \gg 1} \approx U_d - \frac{s}{2}\langle x(t) \rangle \tag{34}$$

Notably, this equation of motion is diagonal and can be easily solved analytically, as is the case for all higher moments of the SFS for alleles under strong additive selection.

Analytic calculation of the trajectory of the mutation burden for recessive selection

Here we are interested in the motion of the first moment $\langle x(t) \rangle$ of the distribution $\phi(t)$ after re-expansion from the bottleneck. First, we consider the equation of motion given by Equation (13), which is derived above. We repeat it here for the convenience of the reader.

$$\partial_t \langle x(t) \rangle \approx U_d - s\langle x(t)^2 \rangle \tag{35}$$

Since the time scale on which the mutation burden rises to a maximum is shorter than the time scale of drift, we can imagine rescaling time by the effective population size $2N_0$ and then working in the perturbative regime $t \ll 1$. This allows us to Taylor expand near $t = 0$ to understand the motion of the burden at early times immediately after the bottleneck. We later determine all of the moments used below and see sufficient subsequent suppression to validate this expansion.

$$\partial_t \langle x(t) \rangle \approx U_d - s\left[\langle x(0)^2 \rangle + t\partial_t \langle x(t)^2 \rangle|_{t=0} + \frac{t^2}{2}\partial_t^2 \langle x(t)^2 \rangle|_{t=0} + O(t^3)\right] \tag{36}$$

To understand the time dependence of $\langle x^2 \rangle$, we analyze the next moment in the same fashion as employed for the first moment, as described in the previous appendix and given in Equation (30).

$$\partial_t \langle x^2 \rangle \approx \frac{U_d}{2N_0} - 2s\langle x^3 \rangle + \frac{2}{4N_0}\langle x \rangle \tag{37}$$

Note that these moments and all higher moments have a non-negligible contribution from the diffusion term in the forward equation.

We model a single-generation bottleneck as a single-generation downsampling of $2N_B$ chromosomes out of the original population of $2N_0$ chromosomes. We can approximately compute $\langle x^2 \rangle$, $\langle x^3 \rangle$, and higher moments if desired, immediately after bottleneck sampling (denoted "$after$") since we have an integral form for $\phi_B(x)$ given by appropriately scaling k in terms of x in Equation (12). Here, ϕ_0 represents the initial pre-bottleneck site frequency spectrum, and the nth moment of this distribution is represented as $\langle x^n \rangle_0$.

$$\langle x^2 \rangle_{after} = \frac{1}{(2N_B)^2} \sum_k k^2 \binom{2N_B}{k} \int dx\ (1-x)^{2N_B-k} (x)^k \phi_0(x) \tag{38}$$

The exchanging the order of the integral and the sum, the sum can be computed directly as a function of x corresponding to the second non-central moment of the binomial distribution. One can compute $\langle x^3 \rangle$ completely analogously.

$$\langle x^3 \rangle_{after} = \frac{1}{(2N_B)^3} \sum_k k^3 \binom{2N_B}{k} \int dx\ (1-x)^{2N_B-k} (x)^k \phi_0(x) \tag{39}$$

The first three non-central moments of the binomial distribution are as follows:

$$\mu'_1 = 2N_B x \tag{40}$$
$$\mu'_2 = 2N_B x(1-x) + (2N_B)^2 x^2 \tag{41}$$
$$\mu'_3 = 2N_B x(1-x)(1-2x) + 3(4N_B^2 x^2(1-x) + 8N_B^3 x^3) - 16N_B^3 x^3. \tag{42}$$

In the limit $N_B \gg 1$, the second and third moments are well approximated by the following expressions.

$$\mu'_2 \approx 2N_B x + (2N_B)^2 x^2 \tag{43}$$
$$\mu'_3 \approx 2N_B x + 3(2N_B)^2 x^2 + (2N_B)^3 x^3 \tag{44}$$

From this we can directly compute the sum in Equations (38) and (39).

$$\begin{aligned}\langle x^2 \rangle_{after} &= \frac{1}{(2N_B)^2} \int dx\ \mu'_2\ \phi_0(x) \\ &= \int dx \left(\frac{x}{2N_B} + x^2 \right) \phi_0(x) \\ &= \frac{\langle x \rangle_0}{2N_B} + \langle x^2 \rangle_0 \end{aligned} \tag{45}$$

For the third moment, we find the following expression.

$$\begin{aligned}\langle x^3 \rangle_{after} &= \frac{1}{(2N_B)^3} \int dx\ \mu'_3\ \phi_0(x) \\ &= \int dx \left(\frac{x}{(2N_B)^2} + \frac{3x^2}{2N_B} + x^3 \right) \phi_0(x) \\ &= \frac{\langle x \rangle_0}{(2N_B)^2} + \frac{3\langle x^2 \rangle_0}{2N_B} + \langle x^3 \rangle_0 \end{aligned} \tag{46}$$

The third moment is relevant in that it allows us to approximately compute the time dependence of the second moment immediately after re-expansion.

$$\begin{aligned}
\partial_t \langle x^2 \rangle_{after} &\approx \frac{U_d}{2N_0} - 2s\langle x^3 \rangle_{after} + \frac{\langle x \rangle_{after}}{2N_0} \\
&= \frac{U_d}{2N_0} - 2s\left(\frac{\langle x \rangle_0}{(2N_B)^2} + 3\frac{\langle x^2 \rangle_0}{2N_B} + \langle x^3 \rangle_0\right) + \frac{\langle x \rangle_0}{2N_0}
\end{aligned} \tag{47}$$

All of the $\langle x^m \rangle_0$ moments can be computed from the initial distribution, determining the Taylor expanded expression in Equation (36) explicitly. These integrals are well approximated in the limit $N_0 s \gg 1$, as described in a following appendix. We calculate the integrals using this approximation and express the first three moments below, the first two of which were described originally in [34].

$$\begin{aligned}
\langle x \rangle_0 &\sim \frac{U_d\sqrt{4N_0}}{\sqrt{s}} \\
\langle x^2 \rangle_0 &\sim \frac{U_d}{s} \\
\langle x^3 \rangle_0 &\sim \frac{U_d}{s\sqrt{4N_0 s}}
\end{aligned} \tag{48}$$

Additionally, we are working under the approximation of a relatively short bottleneck, such that $\langle x \rangle_{after} \approx \langle x \rangle_0 + U_d T_B \approx \langle x \rangle_0$. Corrections can easily be computed to determine the T_B dependence, if desired. Plugging these in, we can gauge the order of magnitude and sign of the initial contributions to the motion of the mutation burden.

$$\begin{aligned}
\frac{\partial_t \langle x^2 \rangle_{after}}{U_d} &\sim \frac{1}{2N_0} - 2\left(\frac{\sqrt{4N_0 s}}{4N_B^2} + \frac{3}{2N_B} + \frac{1}{\sqrt{4N_0 s}}\right) + \frac{2}{\sqrt{4N_0 s}} \\
&\sim -\frac{\sqrt{4N_0 s}}{2N_B^2} - \frac{3}{N_B}
\end{aligned} \tag{49}$$

Note that the $N_0^{-\frac{1}{2}}$ terms exactly cancel in the previous equation and that we have suppressed $O(N_0^{-1})$ corrections. Putting these results together, we integrate Equation (36) to find the following time dependence $\langle x(t) \rangle$.

$$\langle x(t) \rangle \approx \langle x \rangle_0 + U_d t - st\langle x^2 \rangle|_{t=0} - s\left(\frac{t^2}{2}\right)\partial_t \langle x^2 \rangle|_{t=0} + O(t^3) \tag{50}$$

Here the integration constant is simply the initial first moment immediately after re-expansion (which is well approximated by $\langle x \rangle_{after} = \langle x \rangle_0$ in the case of a strong single-generation bottleneck). We substitute our computed value from Equations (48) and (49) in the above equation to compute the time dependence of the mutation burden $\langle x(t) \rangle$.

$$\frac{\langle x(t) \rangle}{U_d} \sim \sqrt{\frac{4N_0}{s}}\left(1 - \frac{st}{2N_B}\right) + \frac{st^2}{2N_B}\left(\frac{\sqrt{4N_0 s}}{2N_B} + 3\right) + O(t^3) \tag{51}$$

At this point, we generalize to multi-generation, but low intensity bottlenecks with the substitution $\frac{1}{2N_B} \rightarrow I_B \equiv \frac{T_B}{2N_B}$. By doing this we have matched the bottleneck intensity to that of a more extreme, but single-generation bottleneck. The time dependence of the mutation burden can be approximated as follows.

$$\langle x(t)\rangle \sim \langle x\rangle_0 \left(1 - stI_B + st^2 I_B \left(sI_B + 3\sqrt{\frac{s}{4N_0}}\right)\right) \tag{52}$$

From this we can easily compute the time dependent form $B_R(t) = \frac{\langle x\rangle_0}{\langle x(t)\rangle}$.

$$B_R(t) \sim \left(1 - stI_B + st^2 I_B \left(sI_B + 3\sqrt{\frac{s}{4N_0}}\right)\right)^{-1} \tag{53}$$

This quadratic time dependence allows us to find extrema. Note that the inclusion of higher order contributions allows for a more accurate temporal dependence $\langle x(t)\rangle$, however this is somewhat unnecessary to understand the dominant behavior of the curve. Concentrating just on this second order expansion in t, we find that the curve first drops from its initial value $\langle x(0)\rangle = \frac{U_d\sqrt{4N_0}}{\sqrt{s}}$, quickly reaches a minimum, and is then brought back up by the positive second order term. The location of the minimum can be found approximately by solving the following equation.

$$\partial_t \langle x(t_{min})\rangle = 0 = -I_B\sqrt{4N_0 s} + I_B 2st_{min}\left(I_B\sqrt{4N_0 s} + 3\right) \tag{54}$$

$$t_{min} \sim \frac{\sqrt{\frac{4N_0}{s}}}{\left(2I_B\sqrt{4N_0 s} + 6\right)} \sim \frac{1}{2}\left(sI_B + 3\sqrt{\frac{s}{4N_0}}\right)^{-1} \tag{55}$$

As expected, the second derivative is positive at this extremum, implying a local minimum.

$$\partial_t^2 \langle x(t_{min})\rangle = 2sI_B\left(I_B\sqrt{4N_0 s} + 3\right) > 0 \tag{56}$$

Plugging t_{min} into our expression for $\langle x(t)\rangle$, we find the approximate magnitude of the mutation burden at this minimum.

$$\langle x(t_{min})\rangle \sim \langle x\rangle_0 \left(1 - \left(4 + \frac{12}{I_B\sqrt{4N_0 s}}\right)^{-1}\right) \tag{57}$$

We have factored out $\langle x\rangle_0 \sim \frac{\theta_0}{\sqrt{4N_0 s}}$ here since it allows for easier calculation of $B_R \equiv \langle x\rangle_0/\langle x\rangle$ below. Thus, in the limit $N_0 s \gg 1$ employed to approximate $\langle x\rangle_0$, we find the following minimum value for the average number of recessive deleterious mutations per genome following a bottleneck.

$$\langle x(t_{min})\rangle \sim \theta_0 \left(\frac{1}{\sqrt{4N_0 s}} - \frac{1}{\left(4\sqrt{4N_0 s} + 12/I_B\right)}\right) \tag{58}$$

From this expression, we can immediately calculate the peak value for the B_R statistic as follows.

$$\begin{aligned} B_R(t_{min}) &\equiv \frac{\langle x \rangle_0}{\langle x(t_{min}) \rangle} \\ &\sim \left(1 - \left(4 + \frac{12}{I_B\sqrt{4N_0 s}}\right)^{-1}\right)^{-1} \\ &\sim \frac{4I_B\sqrt{4N_0 s} + 12}{3I_B\sqrt{4N_0 s} + 12} \end{aligned} \tag{59}$$

We note that in the limit $N_B \gg \sqrt{N_0 s} > 1$ of a low intensity bottleneck ($I_B^{-1} \gg \sqrt{N_0 s}$ for an extended bottleneck), which is biologically relevant for many founder's events in humans, these results simplify as follows. The time dependence of the mutation burden for the founded population is given by,

$$\frac{\langle x \rangle_{after}}{U_d} \sim \frac{\sqrt{4N_0}}{\sqrt{s}}(1 - stI_B) + 3st^2 I_B. \tag{60}$$

This can be used to obtain the functional dependence of $B_R(s, t)$.

$$B_R(t) \sim \left(1 - stI_B + st^2 I_B 3\sqrt{\frac{s}{4N_0}}\right)^{-1} \tag{61}$$

In the limit $I_B^{-2} \gg N_0 s$, the peak response $B_R(t_{min})$ occurs at a time,

$$t_{min} \sim \frac{1}{6}\sqrt{\frac{4N_0}{s}}, \tag{62}$$

and takes the following approximate functional form.

$$B_R(t_{min}) \sim \left(1 - \frac{I_B\sqrt{4N_0 s}}{12}\right)^{-1} \tag{63}$$

We use this expression to compare to simulations in this regime of interest, with the understanding that it breaks down at relatively large bottleneck intensities.

Distribution of selective effects

For a distribution of s effects, the s of maximum effect on B_R is dependent on the time since the bottleneck as given in Equation (23). This describes the transient shift of the elevated load ratio towards smaller s values. To determine the total ${B_R}^{observed}$ at the time of observation t_{obs}, one must integrate over all s values present in the population. This assumes that distinct s classes for recessively acting deleterious alleles can be thought to behave independently in a well mixed, freely recombining diploid population. The distribution of selective effects for de novo mutations, $\rho(s)$, provides the appropriate

weight associated with each class of selective effects, as the mutation rate for mutations of selective effect s is given by $U_d \rho(s)$. Assuming a static distribution of selective effects, we can calculate the observed load ratio at t_{obs}. For a given population, the observed mutation burden $\langle x \rangle^{obs}$ at the time of observation is the mutation burden for each class of selected effects averaged over their representative fraction of new mutations into the population.

$$\langle x(t) \rangle^{obs} = \int ds\, \rho(s)\, \langle x(s,t) \rangle \tag{64}$$

This is true for both the equilibrium and founded populations, allowing us to compute the observed burden ratio ${B_R}^{obs}$ as follows.

$${B_R}^{obs}(t_{obs}) = \frac{\langle x \rangle^{obs}_{eq}}{\langle x \rangle^{obs}_{founded}} = \frac{\int ds\, \rho(s)\, \langle x(s,t_{obs}) \rangle_{eq}}{\int ds\, \rho(s)\, \langle x(s,t_{obs}) \rangle_{founded}} \tag{65}$$

The largest contribution to the load ratio at time t_{obs} occurs at some effective s_{max}, denoted by $s^{observed}_{max}$. The distinction here is that, although s_{max} may have the largest mutation burden, it may occupy only a small fraction of the mutations present in the population when weighted by $\rho(s)$ and thus have a reduced effect on the observed burden ratio ${B_R}^{obs}$. The maximum contribution to the mutation burden s^{obs}_{max} satisfies the following constraint.

$$\partial_s \left(\rho(s) \langle x(s,t) \rangle_{founded} \right) |_{s^{obs}_{max}} = 0 \tag{66}$$

Although we remain agnostic to the distribution of selective effects in the present work, we mention that the model of an exponentially decaying distribution $\rho(s) \sim e^{-\gamma s}$ is somewhat popular in the literature for theoretical, experimental, and aesthetic reasons. As a result, the introduction of such a distribution (or more generally any monotonically decaying distribution) would produce an s^{max}_{obs} value in the range,

$$s_{max} > s^{max}_{obs} \geq 1/2N_0. \tag{67}$$

The selective effect for which the observed change to the load ratio $B_R(t_{obs})$ is maximized has suppressed signal relative to slightly lower s values. This is due to the effective rarity of high s mutations in the population, as they both are introduced at a lower rate $\rho(s_{large}) < \rho(s_{small})$ and are being more efficiently purged from the population due to selection. This indicates that the elevated load ratio $B_R(t_{obs})$ may be most readily observed in the data by looking at mutations with low to intermediate selective effects, rather than those with highest effect. Additionally, we note that the corrected equilibration time for the distribution of effects is given by the time constant associated with s_{obs}.

Most generally, the mutation burden will be comprised of a combination of alleles with varied selective effects and dominance coefficients. Treatment of alleles with intermediate dominance coefficients is discussed below. We can generalize our observed burden ratio as follows.

$${B_R}^{obs}(t_{obs}) = \frac{\langle x \rangle^{obs}_{eq}}{\langle x \rangle^{obs}_{founded}} = \frac{\int dh \int ds\, \rho(s,h)\, \langle x(s,h,t_{obs}) \rangle_{eq}}{\int dh \int ds\, \rho(s,h)\, \langle x(s,h,t_{obs}) \rangle_{founded}} \tag{68}$$

General dominance coefficient and distributions of coefficients

The analysis above presumes that deleterious mutations act with a single average selective effect, either purely additively or purely recessively. One can extend our analysis to the case of partial dominance with a general coefficient $1/2 \geq h \geq 0$, with extreme values corresponding to additivity and recessivity, respectively. We ask at what value of h does the change from $B_R < 1$ to $B_R > 1$ occur. This critical value at some intermediate dominance coefficient $h = h_c$ is of practical interest, as our statistic only has sensitivity to detect whether the average dominance coefficient of a set of alleles lies above or below this critical value. The Kolmogorov equation is easily generalized to include a general dominance coefficient.

$$\begin{aligned}\partial_t \phi(x,t) &= 2NU_d\, \delta\left[x - \frac{1}{2N}\right] + \frac{1}{4N}\partial_x^2(x(1-x)\phi) \\ &\quad + sh\partial_x(x(1-x)\phi(x,t)) + s(1-2h)\partial_x(x^2(1-x)\phi(x,t)) \end{aligned} \tag{69}$$

As detailed in the appendix above, we can use this equation to describe the dynamics of the mutation burden.

$$\partial_t \langle x \rangle \approx U_d - s_A \langle x \rangle - s_R \langle x^2 \rangle \tag{70}$$

Here we have defined $s_A \equiv sh$ and $s_R \equiv s(1-2h)$ for convenience, and taken the strong selection limit in the initial and final population, such that both $2N_0 s_A \gg 1$ and $\sqrt{2N_0 s_R} \gg 1$ are satisfied. In this limit, one can compute the dynamics of the moments after a short bottleneck with completely relaxed selection in complete analogy to the recessive case described above. The perturbative dynamics immediately after re-expansion from the bottleneck are well described by the following Taylor expansion.

$$\begin{aligned}\langle x(t) \rangle &\approx \langle x(0) \rangle + t\,\partial_t \langle x(t) \rangle|_{t=0} + \frac{t^2}{2}\,\partial_t^2 \langle x(t) \rangle|_{t=0} + O(t^3) \\ &\approx \langle x(0) \rangle + t\left(U_d - s_A\langle x \rangle - s_R \langle x^2 \rangle\right)|_{t=0} - \frac{t^2}{2}\left(s_A \partial_t \langle x \rangle + s_R \partial_t \langle x^2 \rangle\right)|_{t=0} + O(t^3) \end{aligned} \tag{71}$$

The critical value occurs when $B_R = 1$, such that $\langle x(t) \rangle_{founded} = \langle x \rangle_0$, providing the following time dependent condition.

$$(\langle x(0) \rangle - \langle x \rangle_0) + t\left(U_d - s_A\langle x \rangle - s_R \langle x^2 \rangle\right)|_{t=0} - \frac{t^2}{2}\left(s_A \partial_t \langle x \rangle + s_R \partial_t \langle x^2 \rangle\right)|_{t=0} \approx 0 \tag{72}$$

As described above, this can be expressed in terms of the moments of the initial distribution $\langle x^n \rangle_0$. The values of s_A, s_R, and all of the moments of the initial distribution are a function of the dominance coefficient h, such that the solution to the above equation provides the critical value h_c. Given the exponential dependence of the initial distribution $\phi_0(x)$ on h, this equation is generally transcendental and thus requires a numerical solution.

Notably, the solution $h_c(t)$ is an inherently time dependent quantity. The additive response is largely due to accumulation of mutations due to relaxed selection during the bottleneck with subsequent decay after re-expansion. In contrast, the recessive response occurs largely after re-expansion due to the purging of newly formed deleterious homozygotes. As a result, at very early times the critical value occurs close to pure recessivity

such that $h_c(t \to 0) \sim 0$, since $B_R < 1$ for even partially additive alleles at this time. The purely additive case equilibrates far more quickly than the recessive case ($t^A_{relax} \propto s^{-1}$ and $t^R_{relax} \propto s^{-1/2}$), such that purely additive alleles become distinguishable from all other cases with even minor excess selection on homozygotes at late times. After this time, nearly additive modes begin to decay, such that there is a breakdown in the definition of h_c since multiple values satisfy the constraint $\langle x(t)\rangle_{founded} = \langle x\rangle_0$. After additive alleles have re-equilibrated, partially recessive alleles remain detectable in times $t^R_{relax} > t > t^A_{relax}$, with the strongest signal coming from purely recessive alleles at $t \geq t_{min} \propto \sqrt{4N_0/s}$. This behavior is summarized in **Figure S1**.

As discussed in the previous section, a distribution of dominance coefficients can be incorporated into the analysis as follows.

$$\langle x\rangle^{obs} = \int dh \int ds\ \rho(s,h)\ \langle x(s,h,t_{obs})\rangle \tag{73}$$

Convolution of multiple dominance coefficients can dilute the recessive signal, as additive and weakly recessive alleles ($h \gtrsim h_c \sim 0.25$) may respond in the opposite direction. As shown by both analytics and simulations, the magnitude of the additive signal is weaker than that for strongly recessive alleles, since it is at most linear in the accumulation of deleterious alleles during the bottleneck and exponentially decays in time. For the present purposes, let us approximate the additive response as roughly the same as that of neutral alleles, as their contribution likely does not significantly deviate from the equilibrium value (consistent with $s = 0$). We have chosen $h_c \sim 0.25$ to be consistent with the Out of Africa value, however a similar analysis could be performed more generally. We can then ask what fraction of new mutations, ϵ_{mut}, fall in the quasi-recessive regime ($h \lesssim h_c \sim 0.25$) that responds nontrivially to the bottleneck.

$$\epsilon_{mut} = \int_0^{h_c \approx 0.25} dh \int_0^1 ds\ \rho(h,s) \tag{74}$$

We may also postulate that the dynamics are driven by segregating alleles, rather than new mutations, and ask what fraction of segregating alleles in an initially equilibrium population are quasi-recessive. This fraction ϵ_{seg} can be computed analagously.

$$\epsilon_{seg} = \frac{\int_0^{h_c \approx 0.25} dh \int_0^1 ds\ \rho(h,s) \int_0^1 dx\ \phi_{eq}(h,s,x)}{\int_0^{h_c \approx 0.5} dh \int_0^1 ds\ \rho(h,s) \int_0^1 dx\ \phi_{eq}(h,s,x)} \tag{75}$$

Here ϕ_{eq} is the SFS (initially in equilibrium), given in Equation 1, and we note that since the SFS is not normalized, we must divide by the total number of segregating mutations. If this fraction is relatively small (perhaps on the order of $\epsilon_{seg} \ll 0.1$) significant dilution occurs, and deviation from $B_R \sim 1$ may not be observable.

Substantial literature has been devoted to the parameterization and quantification of the distribution of dominance coefficients, as elaborated in the introduction of our paper. In particular, the relationship between selective effect and dominance coefficients remains an active field of interest [4, 6, 7, 9, 10, 11, 56]. Estimations of this relationship come exclusively

from model organisms, as established methods require crossing and/or propagation over several generations [7, 11]. In the case of humans and other macroscopic, long lived organisms, these techniques are infeasible. Thus estimates of parameters derived from model organisms may or may not be appropriate to describe comparable quantities in humans, and we proceed with a general parameterization, acknowledging that existing estimates perhaps provide a useful guide.

We split the joint distribution $\rho(h,s)$ into the DFE $\rho(s)$ and a conditional distribution $H(h|s)$ that describes the relationship between selective effects and dominance coefficients. Given the accepted belief of an inverse relationship, we choose an exponential parameterization as described in [7].

$$h = \frac{e^{-\alpha s}}{2} \tag{76}$$

Here α is an organism-specific constant that has been well studied in flies, where a value of $\alpha_{Drosophila} \approx 13$ is suggested in [7]. This parameterization describes only the mean dependence, rather than also fitting the variance, however for the present purposes this is sufficient. The conditional distribution can then be expressed in terms of a delta function as follows.

$$H(h|s) \approx \delta\left(2h - e^{-\alpha s}\right) = \frac{\alpha e^{-\alpha s}}{2}\,\delta\left(s + \frac{\log(2h)}{\alpha}\right) \tag{77}$$

Since h is a positive semidefinite quantity, the second delta function representation is equivalent to the first, and will prove easier to work with. Parameterizing the DFE as an exponential with mean selective effect $\bar{s}$ for analytic simplicity, we write down the joint distribution.

$$\rho(h,s) = H(h|s)\,\rho(s) \approx \delta\left(s + \frac{\log(2h)}{\alpha}\right)\frac{\alpha e^{-\alpha s}}{2}\,\frac{e^{-s/\bar{s}}}{\bar{s}} \tag{78}$$

In the case of new mutations, we can integrate this density directly to find the desired fraction ϵ_{mut}.

$$\begin{aligned}
\epsilon_{mut} &= \int_0^{h_c \approx 0.25} dh \int_0^1 ds\,\delta\left(s + \frac{\log(2h)}{\alpha}\right)\frac{\alpha e^{-s(\alpha+1/\bar{s})}}{2\bar{s}} \\
&= \frac{\alpha}{2\bar{s}}\int_0^{h_c \approx 0.25} dh\;(2h)^{1+1/\alpha\bar{s}} \\
&\sim \frac{\alpha}{\bar{s}\left(1+\frac{1}{\alpha\bar{s}}\right)(2)^{4+1/\alpha\bar{s}}}
\end{aligned} \tag{79}$$

For segregating mutations, we can numerically ascertain the analogous parameter dependence $\epsilon_{seg}^{equil}(\alpha,\bar{s})$ using an equilibrium SFS given in Equation (1).

Requiring a substantial fraction of new mutations to be quasi-recessive amounts to a transcendental inequality of the following form.

$$0.1 \lesssim \frac{\alpha}{\bar{s}\left(1+\frac{1}{\alpha\bar{s}}\right)(2)^{4+1/\alpha\bar{s}}} \tag{80}$$

Numerical solutions to this equation can provide a bound on α for a given average selective effect $\bar{s}$. For humans, the parameter α is unknown, and most estimates of $\bar{s} \in [0.0001, 0.001]$

explicitly assume additivity in their method of inference. Naively taking this range, one can numerically show that on the weak selection end ($\bar{s} \sim 0.0001$) the rough bound requires $\alpha \gtrsim 500$, and on the strong selection end ($\bar{s} \sim 0.001$) the bound requires $\alpha \gtrsim 50$. These values are significantly higher than the estimate for flies ($\alpha \sim 10$), implying that, on the whole genome level, weak effect mutations substantially dilute the measured B_R making observation of recessive variants unlikely unless $\alpha_{human} \gtrsim 50$. By restricting to segregating sites in an equilibrium population, a bound can be numerically derived using $\epsilon_{seg}^{equil} \gtrsim 0.1$. This bound is substantially stronger, such that for $\bar{s} \sim 0.001$, the allowed values are roughly $\alpha_{human} \gtrsim 1000$.

Based on numerical solutions of Equation (80) over a large range of parameters (and the analogous integral equation for segregating mutations), the bound appears to be monotonic and can be very roughly approximated as $\alpha \gtrsim 0.05/\bar{s}$ for new mutations and roughly $\alpha \gtrsim 1/\bar{s}$ for segregating mutations in a quasi-equilibrium population in mutation-selection-drift balance. We note that in real human populations, for example in both Africans and Europeans, recent exponential expansion results in the accumulation of many segregating rare deleterious mutations at sub-drift frequencies such that the SFS deviates substantially from Equation (1) at the low frequency end. This can drive the true fraction of segregating quasi-recessive sites in an expanding population towards the larger fraction associated with new mutations, such that $\epsilon_{seg}^{exp.\ growth} \to \epsilon_{mut}$, where $\epsilon_{mut} > \epsilon_{seg}^{exp.\ growth} > \epsilon_{seg}^{equil}$. As a result, the relevant bound in a population that experienced recent exponential growth may be closer to $\alpha \gtrsim 0.05/\bar{s}$ for a set of alleles with average selection strength $\bar{s}$, however the exact bound is highly dependent on the recent demographic history of the population.

The dependence of the bound on the strength of selection indicates that deviation from $B_R = 1$ is likely to be observable for the strongest effect mutations. In the case that humans and flies have similar joint distribution of selective effects and dominance coefficients, such that $\alpha_{human} \sim \alpha_{Drosophila} \sim 10$, we find that $\bar{s} \sim 0.01$ may be sufficient to observe strong deviation from $B_R \sim 1$ due to the prevalence of rare (highly deleterious) partially recessive variants in a recently exponentially expanded population. This is consistent with our observations of $B_R > 1$ for a set of predicted deleterious variants in medically important genes, and highlights the usefulness of recessive Mendelian disease genes for demonstrative purposes and potential future applications.

Additionally, the present analysis indicates that for diploid organisms with very large $\alpha \gg \alpha_{Drosophila}$, such that a large fraction of variants are at least partially recessive ($h < h_c$), it may be possible to create a more detailed map of the average dominance coefficients for specific genes using this method. Such an organism would also allow for detection of $B_R > 1$ on the whole genome level, such that sequencing of low coverage genomes may be sufficient to bound α, provided a population split, bottleneck, and expansion occurred relatively recently. We also note that the present analysis details results appropriate for the human Out of Africa bottleneck (where $h_c \sim 0.25$) that ended roughly 1000 generations in the past. Distinct demographic events could provide different, potentially stronger bounds on α, and likely warrant a similar analysis for different h_c values.

Long bottleneck limit

In the case of a long bottleneck of duration $T_B \sim O(2N_B)$ generations, the bottlenecked population has had sufficient time to equilibrate into mutation-selection-drift balance with the new population size $2N_B$. The site frequency spectrum can be written in the same form given by Equation (1). In the case of recessive variation, we find the following form during the bottleneck.

$$\phi(x) = \theta_B \frac{e^{-2N_B s x^2}}{x(1-x)} \left[1 - \frac{\int_0^x e^{2N_B s x^2}}{\int_0^1 e^{2N_B s x^2}} \right] \tag{81}$$

Here we have defined $\theta_B \equiv 4N_B U_d$. In the limit $N_b s \gg 1$, this can be written approximately as follows.

$$\phi(x) \approx \theta_B \frac{e^{-2N_B s x^2}}{x} \tag{82}$$

Immediately after re-expansion from the bottleneck, the first three moments of this distribution can be easily calculated using the Gaussian integrals described in an appendix below. These can be substituted into the Taylor expanded time dependent form for $\partial_t \langle x(t) \rangle$ in Equation (14) to analyze the dynamics and solve for the functional dependence of the B_R statistic.

For analysis of bottlenecks of intermediate length, a full non equilibrium description is required, but this can be well approximated by analytically patching the solutions given by the single-generation and long bottleneck limits.

Exponential expansion and more general geometries

Exponential expansion is a general feature of many natural populations, particularly after a founding event, motivating the generalization of our analysis to such cases. In this work, we describe the transient behavior of $B_R(s,t)$, and the s values that are favored as time progresses. As a result, this behavior is extremely sensitive to exponential expansion, for example, as opposed to the simple square bottleneck model described above. In the most general case, we may have a general time dependence for the population size after the bottleneck, which sensitively effects the s values for which the burden ratio B_R is largest. For an explanatory example, we will model the immediate exponential inflation of the size of both the founded and equilibrium populations after re-expansion from the bottleneck.

$$N_f(t) \sim N_0 e^{t/a} \tag{83}$$

We rescale time by the population size $t^I \equiv t\frac{2N_0}{2N_f} = te^{-t/a}$, yielding exponentially slowed "inflated" time in the decelerated frame of the fixed population size. In this rescaled frame we can analyze the shift of the transient peak of the load ratio (in inflated time) ${B_R}^I(s^I, t^I)$ by plugging our new scaled time into Equation (23).

$$s^I_{max} \sim \frac{2N_0 e^{2t/a}}{10t^2} \tag{84}$$

Note that this factor of N_0 refers to the initial population size prior to the bottleneck, and does not get rescaled due to the inflating population size. Taylor expansion of the exponential demonstrates that there is a perturbative crossover at time $t \sim 2a$.

$$s^I_{max} \propto \left(\frac{1}{t^2} + \frac{2}{at} + \frac{2^2}{2a^2} + \frac{2^3 t}{6a^3} + ... \right) \tag{85}$$

When $t \sim a$, the third term in the expansion, initially the quadratic term of the exponential, finally begins to dominate over the second term in the expansion. At this point positive powers become technically relevant in the perturbative expansion. This is the transition between the initial transient decrease in s_{max} and the exponential freezing out of the rapidly decaying large s components of B_R. At this time, the maximum of the load ratio is given by,

$$s_{max} \sim \frac{2N_0 e^2}{10a^2} \approx \frac{2N_0}{a^2}. \tag{86}$$

For very rapid inflation, a is small, indicating that the dominant modes in B_R still exist at high s values, such that $s_{max} \gg 1$. For large $a \gg 1$, corresponding to slow, even adiabatic, expansion, the transient rapidly decays towards smaller s values, such that $s_{max} \ll 1$. Intermediate values are particularly interesting, as the rate of expansion can actually compete dynamically with the transient decay. In this case, any intermediate selection effect may be frozen in, dominating the signature in the burden ratio B_R.

Evaluating Gaussian integrals

The steady state distribution prior to the bottleneck is well approximated by the following form.

$$\phi_0 \approx \theta_0 \frac{e^{-2N_0 s x^2}}{x(1-x)} \tag{87}$$

The decay at large frequencies is made even more rapid by the suppressed terms, so for the present argument this form is sufficient. Computing the first moment of this distribution corresponds to the following integral.

$$\langle x \rangle_0 \approx \theta_0 \int_0^1 dx\, x\, \frac{e^{-2N_0 s x^2}}{x(1-x)} \approx \theta_0 \int_0^1 dx\, \frac{e^{-2N_0 s x^2}}{1-x} \tag{88}$$

For sufficiently large $2N_0 s \gg 1$, the exponential rapidly converges prior to reaching the $x = 1$ upper limit. In this case, in addition to canceling the linear terms in the numerator and denominator, the $(1-x)$ term in the denominator is highly suppressed by the exponential. The first moment can be simply computed as half of a Gaussian integral.

$$\langle x \rangle_0 \approx \theta_0 \int_0^1 dx\, e^{-2N_0 s x^2} \approx \frac{\theta_0}{2} \int_{-\infty}^{\infty} dx\, e^{-2N_0 s x^2} \approx \frac{\theta_0}{2} \sqrt{\frac{\pi}{2N_0 s}} \tag{89}$$

Using the following definition, we can compute the first few moments of interest for the site frequency spectrum $\phi(x)$ of recessive deleterious mutations.

$$\langle x^{n+1}\rangle_0 \propto \int_0^\infty dx\, x^n e^{-\gamma x^2} = \begin{cases} \frac{(n-1)!!}{(2\gamma)^{n/2}}\frac{1}{2}\sqrt{\frac{\pi}{\gamma}} & \text{for even n} \\ \\ \frac{\left(\frac{1}{2}(n-1)\right)!}{2\gamma^{(n+1)/2}} & \text{for odd n} \end{cases} \tag{90}$$

The first few moments are given by the following equations.

$$\langle x\rangle_0 \approx \theta_0 \int_0^1 e^{-2N_0 s x^2} \approx \frac{\theta_0}{2}\sqrt{\frac{\pi}{2N_0 s}} \sim \frac{\theta_0}{(4N_0 s)^{1/2}} \tag{91}$$

$$\langle x^2\rangle_0 \approx \theta_0 \int_0^1 x e^{-2N_0 s x^2} \approx \frac{\theta_0}{4N_0 s} \tag{92}$$

$$\langle x^3\rangle_0 \approx \theta_0 \int_0^1 x^2 e^{-2N_0 s x^2} \approx \frac{\theta_0}{8N_0 s}\sqrt{\frac{\pi}{2N_0 s}} \sim \frac{\theta_0}{(4N_0 s)^{3/2}} \tag{93}$$

$$\langle x^4\rangle_0 \approx \theta_0 \int_0^1 x^3 e^{-2N_0 s x^2} \approx \frac{\theta_0}{2(2N_0 s)^2} \sim \frac{2\theta_0}{(4N_0 s)^2} \tag{94}$$

Simulation curve collapse

Here we extend our analysis of the accuracy of our analytic results by continuing to scrutinize the comparison with our simulation. To represent how the breakdown of our approximation depends on the selective coefficient, we plot a subset of the data labeled by selective effect size s in **Figure S2**. We find generally good agreement between analytic approximations and simulation (represented by a flat line at one on the plots in **Figure S2**). We note that deviations from our analytic scaling occur most substantially when both the selective effect s and bottleneck intensity I_B are large, implying that the correct scaling of a more extended bottleneck involves a correction to the s dependence. This is due to the approximation of neutrality during the bottleneck. Alleles under selection eventually equilibrate during the bottleneck, with faster equilibration times for alleles under strong selection, such that this approximation breaks down for either long bottlenecks or strong selection. The second plot in **Figure S2** represents such a scaling, motivated by analytic dependence of the burden ration on $sqrt(s)$.

Variable definition legend

B_R	Mutation burden ratio.
x	Allele frequency.
s	Magnitude of selection of deleterious alleles.
h	Dominance coefficient for deleterious alleles.
U_d	Per individual deleterious mutation rate.
ϕ	Site frequency spectrum (SFS).
$\langle x \rangle$	Per haploid individual mutation burden.<br>Equivalently, weighted mean of the SFS.
$\langle x^2 \rangle$	Homozygosity.<br>Equivalently, second non-central moment of the SFS.
N_0	Number of diploid individuals in initial (and final) population.
ϕ_0, $\langle x \rangle_0$, $\langle x^2 \rangle_0$	Initial (equilibrium) site frequency spectrum and corresponding initial moments.
ϕ_B, $\langle x \rangle_B$, $\langle x^2 \rangle_B$	Site frequency spectrum and corresponding moments at the end of the bottleneck.
N_B	Number of diploid individuals during bottleneck.
T_B	Duration of bottleneck.
I_B	Bottleneck intensity.
t	Time after end of bottleneck/re-expansion.
t_{min}	Time of minimum mutation burden in founded population after re-expansion in response to population bottleneck.
$B_R(t_{min})$	Maximum mutation burden ratio after re-expansion.
t_{obs}	Time of observation of the burden ratio after re-expansion.
s_{max}	At time of observation, selection strength associated with the maximum value of B_R.<br>Also, most readily observable selection coefficient, given a demography and observation time.
t_{relax}	Maximum relaxation time (re-equilibration time) of B_R for recessive alleles of any selection strength.
h_c	Critical dominance coefficient separating above and below $B_R = 1$.<br>Also, maximum bound for empirically observed recessive alleles.
$\rho(s)$	Distribution of selection strengths.<br>Equivalently, distribution of fitness effects (DFE).
$\rho(s, h)$	Joint distribution of selection strengths and dominance effects.
ϵ_{mut}	Fraction of *de novo* mutations that are recessive ($h \ll 0.5$) necessary to observe a signal in B_R.
ϵ_{seg}	Fraction of segregating mutations that are recessive ($h \ll 0.5$) necessary to observe a signal in B_R.
α	Selection-dominance coupling parameterizing inverse correlation of h and s.
$\bar{s}$	Estimated average selection strength in an exponential DFE.

Additional References

[55] Ewens WJ (2004) Mathematical Population Genetics: I. Theoretical introduction. Vol. 27. Springer, 2004.

[56] Keightley PD (1996) A metabolic basis for dominance and recessivity. Genetics 143:621625.
