# Supplementary material for: Dominance of Deleterious Alleles Controls the Response to a Population Bottleneck
Source: PLoS Genet. 2015 Aug 28;11(8):e1005436. doi: 10.1371/journal.pgen.1005436 (PMC4552954; doi:10.1371/journal.pgen.1005436)
Supplement: S2 Text — Here we describe further details of the analyzed data. Gene sets are discussed in detail, and are included in a supplemental file. We discuss the results for B R per gene set (with corresponding standard errors), and include an analysis of synonymous sites as a negative control for damaging and nonsense sites in these gene sets. (PDF) [file pgen.1005436.s002.pdf]

Text S2. Data Analysis Details

Empirical Evidence for Detection of Recessive Selection

To test whether the B_R statistic is predictive of recessive selection, we compare human data from European populations, known to have undergone a relatively intense bottleneck during the "Out of Africa" event, to African populations that did not experience a founder's event. We analyze exome data from the Exome Sequencing Project (ESP) and validate some of our findings using exome data from the 1000 Genomes Project (1KG)[41, 37]. Specifically, in ESP, we compare the average per haploid mutation burden in 1088 European Americans(EA) with largely European ancestry to 1351 African Americans (AA) with substantial African ancestry. In 1KG, we compare 85 Northern Europeans from Utah (CEU) to 88 Yorubans (YRI). This provides a distribution of gene scores ranging from predicted additive (or dominant) ($B_R < 1$) to predicted recessive ($B_R > 1$), derived from the distinct per gene mutation burdens from each population. We sum these mutation burdens over genes of interest to compute an aggregate B_R score for a given gene set. We use several lists of genes associated with known autosomal recessive diseases to determine whether genes potentially under recessive selection due to disease association show statistically significant signature $B_R > 1$ in the burden ratio statistic. We find evidence suggesting statistical deviation from neutrality (in the recessive direction) in reasonably obtained AR disease gene sets. These results are not carried through to substantially larger gene sets that sample a large fraction of the genome, consistent with previous studies of related statistics on the whole genome level[36, 35]. Additionally, we perform several controls to demonstrate the robustness of these results. Despite substantial admixture in the African Americans sequenced in ESP, leading to decreased power of B_R, we find significant results, one of which is validated by using 1KG, which is known to contain much less admixture, but sequences far fewer individuals.

To compute B_R gene scores, we restrict our analysis to non-synonymous nonsense variants and variants predicted to be damaging using a human-free version of PolyPhen2 [36]. This software was developed to remove bias due to the mixed ancestry of the human reference sequence, and annotates derived alleles based on chimpanzee orthologs. We note that many genes contain no damaging or nonsense variants in either one or both population samples, however on the level of tens of genes, this issue is mitigated. We compute the per-haploid mutation burden for the aggregated subset of variants occurring in the genes of interest. For nonsense and damaging variants, this can be represented simply as the sum of all derived allele frequencies for these variants.

$$\langle x \rangle^{dam} \equiv \sum_i x_i^{dam} \tag{95}$$

This quantity is separately computed in the African and European populations, and compared to produce the burden ratio.

$$B_R{}^{dam} \equiv \frac{\langle x \rangle_{AA}^{dam}}{\langle x \rangle_{EA}^{dam}} \tag{96}$$

We use several lists of genes associated with AR diseases, such that, in the absence of pleiotropy and the presence of purifying selection against these disease phenotypes, we naively expect these genes to act under partial or total recessive selection. First we compile a set of genes from the Human Gene Mutation Database (HGMD) only associated with diseases with "autosomal recessive" in the disease name [38]. We restrict this set to genes with at least 5 disease-associated variants to guarantee sufficient polymorphism and reduce noise in the B_R statistic. This set contains 38 genes that appear in the list of ESP scored genes (44 in 1KG) and is referred to as "HGMD". We use Congenital Hearing Loss as an example of a polygenic, largely recessive disease. We obtained an annotated gene list of AR genes associated with hearing loss from the Laboratory for Molecular Medicine (LMM) [39]. This list contains 30 genes in ESP (37 in 1KG) and is referred to as "Hearing Loss". Notably, this list excludes connexin 26 (GJB2), among other genes, which has additional association with AD hearing loss. Additionally, we assemble a combined list of all genes from HGMD and Hearing Loss, with a total of 60 genes in ESP (72 in 1KG) after removing overlap, referred to as "Combined". To assemble a larger, though noisier gene set, we use all annotated AR genes in the Clinical Genomic Database, referred to as "CGD", which contains 1268 genes in ESP and 1348 genes in 1KG [40].

For each gene set we determine the group B_R score and statistical significance for damaging and nonsense variation, as summarized in **Table S1**. To analyze statistical significance, we compute a one-sided p-value using 10000 bootstrap sampled gene sets of the same size as the gene set of interest. The bootstrap value is computed by sampling n genes from a gene set of n genes with replacement after each sample. The mean B_R is computed on each bootstrapped gene set, such that the variance of this quantity over all 10000 sets estimates the standard error on the mean of the original gene set. We then rank the neutral hypothesis $B_R = 1$ in this list, letting the p-value determine statistical significance of rejection of the null hypothesis. In other words, the p-value represents the significance of deviation from neutrality in the recessive direction. We find that HGMD and HL both deviate from neutrality at the $p < 0.05$ level, as does the combined gene set. For comparison to a known statistic, we show the results of a paired Student t-test measuring deviations of the mean African and mean European per gene mutation burden. The significance pattern is identical to that of the B_R statistic, although the power appears to be reduced. We replicate some of our results from ESP using an independent dataset, 1KG, finding statistical significance in the HGMD disease gene set. As an additional control, we compute B_R using only fourfold degenerate synonymous variants, ${B_R}^{syn} \equiv \langle x \rangle_{AA}^{syn} / \langle x \rangle_{EA}^{syn}$, predicted to behave neutrally, as summarized in **Table S2**. In 1KG, we find statistical significance in ${B_R}^{syn}$ for all genes combined. This result represents only a very slight deviation from ${B_R}^{syn} = 1$, and is not replicated in ESP, potentially indicating spurious significance. This exception could be due to weak selection on synonymous sites, linkage between synonymous variants and other variants under selection, or may simply be a statistical fluctuation. All other gene sets in both data sets show no significance in the $B_R > 1$ direction for fourfold degenerate synonymous sites, such that they provide a negative control for the detection of recessive selection.

Given the statistical significance of two distinct disease gene sets in ESP, one of which that is replicated in 1KG, in combination with the null results in nearly all controls, the data suggestive of the utility of the B_R statistic for identifying alleles under recessive selection.
