# Supplementary material for: Dominance of Deleterious Alleles Controls the Response to a Population Bottleneck
Source: PLoS Genet. 2015 Aug 28;11(8):e1005436. doi: 10.1371/journal.pgen.1005436 (PMC4552954; doi:10.1371/journal.pgen.1005436)
Supplement: S3 Text — Here we present the simulation code for convenience. This can also be found at http://genetics.bwh.harvard.edu/wiki/sunyaevlab/dbalick. (PDF) [file pgen.1005436.s003.pdf]

Text S3. Simulation code.

Here we present the simulation code (written in **C**) used in our paper. This can also be found at http://genetics.bwh.harvard.edu/wiki/sunyaevlab/dbalick.

We note that some included libraries are lab-specific, but perform very basic statistical functions. Interested readers should substitute appropriate routines in this code to produce results. Alternatively, a compiled version is available on the website listed above.

```
/* burden_sim */
/* by David Reich */

#include <nag.h>
#include <nag_stdlib.h>
#include <stdio.h>
#include <stdlib.h>
#include <math.h>
#include <nage04.h>
#include <nicklib.h>

FILE *fout;

void newgravel();
void tennessen();
void lohmueller();
void simplebottle();
void calcstats();
int  drift();
int  mutate();
void printhist();

int totmuts;
int fixedmuts;

#define MX 650000
#define BINS 100

void printhist(int muts,int mat[MX][3],int pop[2])
{
	int i,j,hist[BINS][2],count[2],val;

	for (j=0;j<2;j++) {
		count[j]=0;
		for (i=0;i<BINS;i++) hist[i][j]=0;
		for (i=0;i<muts;i++) {
			val=(int)floor(1.0*BINS*mat[i][j]/pop[j]);
			if (mat[i][j]>0 && mat[i][j]<pop[j]) {
				++hist[val][j];
				++count[j];
			}
		}
	}
	fprintf(fout,"\nlower\tupper\tpop0\tpop1\n");
	for (i=0;i<BINS;i++) {

	fprintf(fout,"%3.2lf\t%3.2lf\t%6.5lf\t%6.5lf\n",1.0*i/BINS,1.0*(i+1)/BINS,1.0*hist
[i][0]/count[0],1.0*hist[i][1]/count[1]);
	}

}

void calcstats(long x,int gen, int speedup1,int speedup2,int burnin1,int burnin2, int
lastgen, double mu, double s, double dom, int mat[MX][3],int pop[2],int muts,int len,int
speed)
{
```

```
	int i,j;
	double sum[2],addburden[2],theta[2],mean[2],pi[2],recburden[2],vv,gval;

	for (j=0;j<2;j++) {
		sum[j]=pi[j]=recburden[j]=addburden[j]=mean[j]=theta[j]=0;
		for (i=0;i<muts;i++) {
			vv=(1.0*mat[i][j]/pop[j])*(1-(1.0*mat[i][1-j]/pop[1-j]));
			if (vv<0)
printf("%d\t%d\t%d\t\t%d\t%d\t%d\t%d\n",gen,j,i,mat[i][j],pop[j],mat[i][1-j],pop[1-j]);
			addburden[j]+=(1.0*mat[i][j]/(1.0*pop[j]))/len;
			gval=(1.0*mat[i][j]*(mat[i][j]-1)/(1.0*pop[j]*(pop[j]-1)))/len;
			if (gval<0) fatalx("ERROR\n");
			recburden[j]+=gval;
			if (mat[i][j]>0 && mat[i][j]<pop[j]) {
				pi[j]+=2.0*mat[i][j]*(pop[j]-
mat[i][j])/(1.0*pop[j]*(pop[j]-1)*len);
				theta[j]+=1.0/((log(1.0*pop[j])+0.577)*len);
				mean[j]+=1.0*mat[i][j]/(1.0*pop[j]);
				++sum[j];
			}
		}
		mean[j]=mean[j]/sum[j];

	}
	fprintf(fout,"%ld\t%d\t%d\t%d\t%d\t%d\t%d\t%d\t%d\t%3.2lf\t%10.9lf\t%4.3lf\t%d\t%d
\t%d\t%d\t%d\t%10.9lf\t%10.9lf\t%10.9lf\t%10.9lf\t%10.9lf\t%10.9lf\t%10.9lf\t%10.9lf\t%10
.9lf\t%10.9lf\n",
		x,gen,gen-lastgen,speedup1,speedup2,-burnin1,-
burnin2,lastgen,len,100000000*mu,s,dom,totmuts,fixedmuts,muts,pop[0]*speed,pop[1]*speed,

	pi[0],pi[1],theta[0],theta[1],mean[0],mean[1],recburden[0],recburden[1],addburden[
0],addburden[1]);
	printf("%d\t%d\t%d\t%7.6lf\t%7.6lf\t%7.6lf\t%7.6lf\n",gen,speed*pop[0],speed*pop[1
],pi[0],pi[1],addburden[0],addburden[1]);
}

void newgravel(int gen,int pop[2],int speed)
{
	int j;
	double p;

	pop[0]=28948;
	if (gen<3880) pop[1]=pop[0];
	if (gen>=3880 && gen<5000) pop[1]=3722;
	if (gen>=5000) pop[1]=(int)floor(2064*exp(0.003857868074807*(gen-5000)));
	 /* end time is 5920 */
	for (j=0;j<2;j++) pop[j]=(int)floor(0.5+1.0*pop[j]/speed);
}

void simplebottle(int gen,int pop[2],int speed)
{
	 int j;
	 double p;

	 pop[0]=pop[1]=28948;
	 if (gen>=3880 && gen<4080) pop[1]=500;
	 for (j=0;j<2;j++) pop[j]=(int)floor(0.5+1.0*pop[j]/speed);
}

void tennessen(int gen,int pop[2],int speed)
{
	 int j;
	 double p;

	 if (gen<5716) pop[0]=28948;
	 if (gen>=5716 && gen<=5920) pop[0]=(int)floor(28948*exp(0.0166*(gen-5716)));

	 if (gen<3880) pop[1]=28948;
	 if (gen>=3880 && gen<5000) pop[1]=3722;
	 if (gen>=5000 && gen<5716) pop[1]=(int)floor(2064*exp(0.00307*(gen-5000)));
	 if (gen>=5716) pop[1]=(int)floor(18900*exp(0.0195*(gen-5716)));
	 /* end time is 5920 */
	 for (j=0;j<2;j++) pop[j]=(int)floor(0.5+1.0*pop[j]/speed);
```

```
}


int drift(int gens,int split,int newpop[2],int oldpop[2],int mat[MX][3], int muts,double
s,double dom)
{
	int i,j;
	double p,q,exp;

//	printf("selection=%8.7lf\n",s);
	for (i=0;i<muts;i++) {
		for (j=0;j<2;j++) {
			if ((j==0) || (j==1 && split==1)) {
				p=1.0*mat[i][j]/oldpop[j];
				q=1-p;

	exp=(p*p*(1+s)+p*q*(1+dom*s))/(p*p*(1+s)+2*p*q*(1+dom*s)+q*q);
				mat[i][j]=ranbinom(newpop[j],exp);
			}
			else mat[i][1]=mat[i][0];
		}
		if (mat[i][0]+mat[i][1]==0) {
			for (j=0;j<3;j++) mat[i][j]=mat[muts-1][j];
			--muts;
		}
		if ((mat[i][0]==newpop[0]) && (mat[i][1]==newpop[1])) {
			for (j=0;j<3;j++) mat[i][j]=mat[muts-1][j];
			--muts;
			++fixedmuts;
		}

	}

	return(muts);
}

int mutate(int split,double param,int pop[2],int mat[MX][3],int gen,int muts)
{
	int newmuts,j,k;

	for (k=0;k<2;k++) {
		if ((k==0) || (k==1 && split==1)) {
			newmuts=(int)ranpoiss(param*pop[k]);
			totmuts+=newmuts;
			if (muts+newmuts>MX) fatalx("overflow\n");
			for (j=muts;j<muts+newmuts;j++) {
				mat[j][k]=1;
				mat[j][1-k]=0;
				if (k==0 && split<1) mat[j][1]=1;
				mat[j][2]=gen;
			}
			muts=muts+newmuts;
		}
	}

	return(muts);
}

void main (int argc, char *argv[])
{
	int
split,lastgen,pops,newmuts,muts,i,j,k,len,newpop[2],oldpop[2],burnin1,burnin2,mat[MX][3],
speedup1,speedup2,speed;
	double mu,s,dom;
	long x;

	lastgen=5921;			// currently set for Gravel model
	mu=atof(argv[1]);		// mutation rate per base pair
	s=atof(argv[2]);		// selection coefficient
	dom=atof(argv[3]);		// dominance coefficient classic h (0.5 is additive,
1 is fully recessive for the new allele)
	len=atoi(argv[4]);		// locus size
```

```
	burnin1=atoi(argv[5]);			// burnin generations for most ancient
burning
	burnin2=atoi(argv[6]);			// burnin generations for more recent burnin
	speedup1=atoi(argv[7]);			// speedup factor which is an integer for
most ancient burnin
	speedup2=atoi(argv[8]);			// speedup factor which is an integer for
more recent burnin

	fout = fopen(argv[9],"w");	// output file name
	fprintf(fout,"seed\tabsolute_gen\tgen_in_past\tspeedup1\tspeedup2\tburnin1\tburnin
2\tenddate\tlocus_length_in_bp\tmu\ts\th\ttotmuts\tfixmuts\tsegmuts\tpop0\tpop1\t");
	fprintf(fout,"pi0\tpi1\ttheta0\ttheta1\tmean0\tmean1\trecburd0\trecburd11\taddburd
0\taddburd1\n");
	x=seednum();
	SRAND(x);
	totmuts=fixedmuts=muts=split=0;
	speed=speedup1;
	for (i=-burnin1;i<=lastgen;i=i+speed) {
		if (i>=3880) split=1;		// split time
		simplebottle(i,newpop,speed);
		muts=drift(i,split,newpop,oldpop,mat,muts,speed*s,dom);
		muts=mutate(split,speed*mu*len,newpop,mat,i,muts);
		if (i>=-(burnin2+1)) speed=speedup2;
		for (j=0;j<2;j++) oldpop[j]=newpop[j];
		if ((i%5000==0 && i<0) || (i%100==0 && i>=0) || (i==lastgen))
calcstats(x,i,speedup1,speedup2,burnin1,burnin2,lastgen,mu,s,dom,mat,newpop,muts,len,spee
d);
	}
	printhist(muts,mat,newpop);
	fclose(fout);

}
```
