# Supplementary figures and images for: Dominance of Deleterious Alleles Controls the Response to a Population Bottleneck
Source: PLoS Genet. 2015 Aug 28;11(8):e1005436. doi: 10.1371/journal.pgen.1005436 (PMC4552954; doi:10.1371/journal.pgen.1005436)

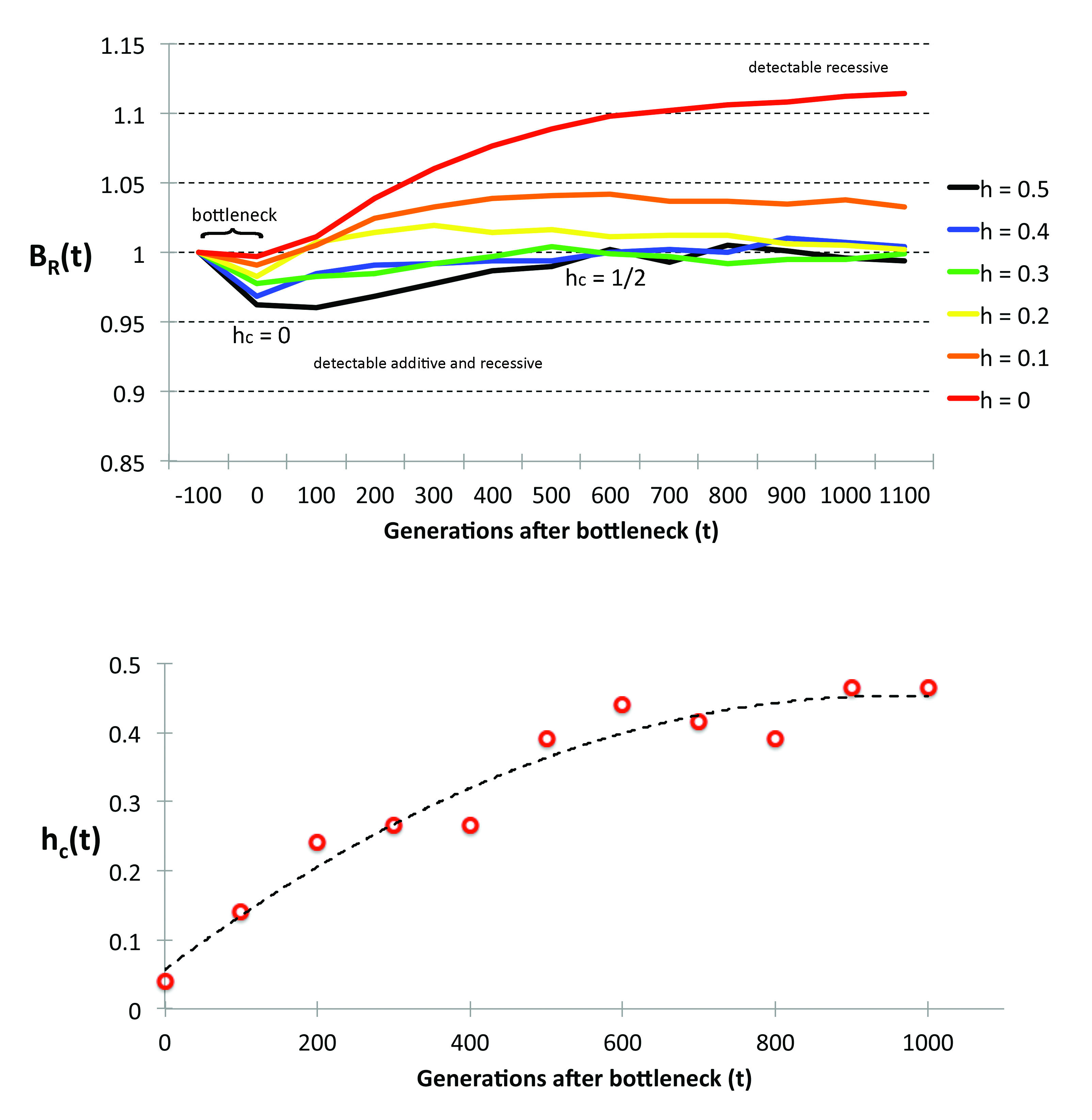

Supplement: S1 Fig — ABOVE: B R is plotted for several values of dominance coefficient h as a function of time after re-expansion from the bottleneck to demonstrate the observable range on either side of the critical dominance coefficient. Additive and recessive alleles are distinguishable when observing at early times prior to re-equilibration due to additive selection. During the equilibration process, the critical value of the dominance coefficient h c(t) at which B R = 1 shifts from near pure recessivity (h c ∼ 0) at early times to near additivity at late times (h c = 1/2). After additive re-equilibration, partially recessive alleles are still detectable (B R > 1) with purely recessive alleles providing the largest signature prior to their eventual equilibration. In this plot 2N 0 = 20000, s = 10−2, T B = 100 and 2N B = 2000 such that I B = 0.05. This qualitative behavior is generic for most parameter values in the short, low intensity bottleneck limit I B ≪ 1, however the time dependence of h c depends sensitively on these parameters.BELOW: The critical dominance coefficient h c is plotted as a function of time. At early times h c ∼ 0 is close to pure recessivity. After re-equilibration of additive alleles, h c ∼ 1/2, such that only partially recessive alleles provide a signature. Any value B R > 1 provides evidence of alleles under partially recessive selection, with the largest contribution coming from purely recessive alleles. (TIFF) [file pgen.1005436.s005.tiff]

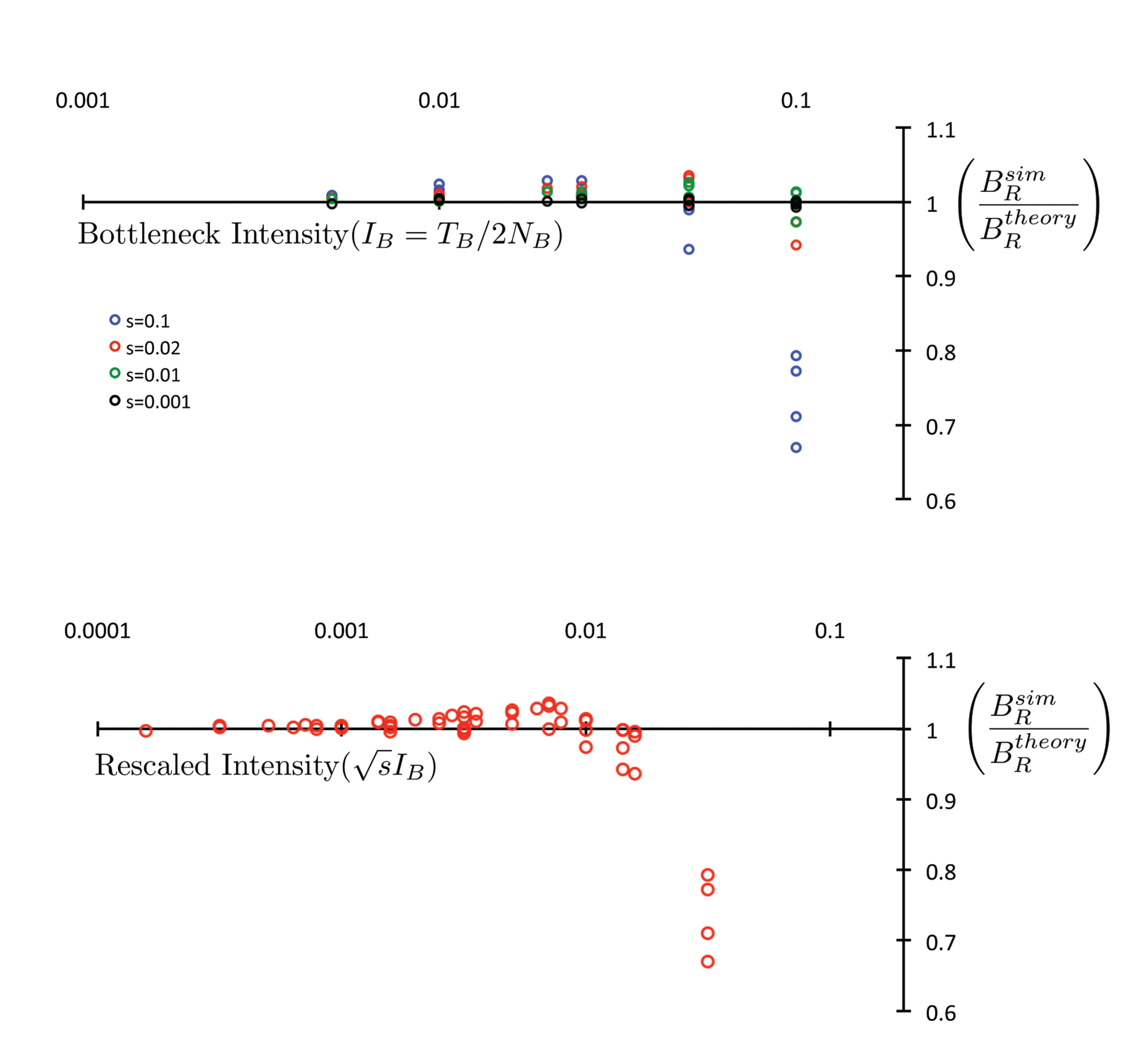

Supplement: S2 Fig — ABOVE: Here we plot a curve collapse for the peak response B R(t min) to compare our analytic description to simulated data. Values near BRsim/BRanalytic=1 validate our analytic description. Deviation from this line represents a breakdown in the proposed scaling as a function of the intensity and selective effect. We find that the collapse is weakly stratified by selective coefficient, even in the range of good agreement at low intensity. Large selective coefficients s = 0.1 deviate fastest, implying a breakdown in the short bottleneck scaling of B R(s). Parameter values of 2N B = 2000, T B = {200,100,50,20}, and s = {0.1,0.02,0.01,0.001} are included on the plot. B R(t min(s)) occurs at different times t min(s) for different selection coefficients.BELOW: BRmin curve collapse is plotted as a function of rescaled intensity sIB to illustrate that breakdown of our theoretical predictions occurs in the limit IB≫1/2N0s, where N 0 is fixed in this collapse for illustrative purposes. (TIFF) [file pgen.1005436.s006.tiff]

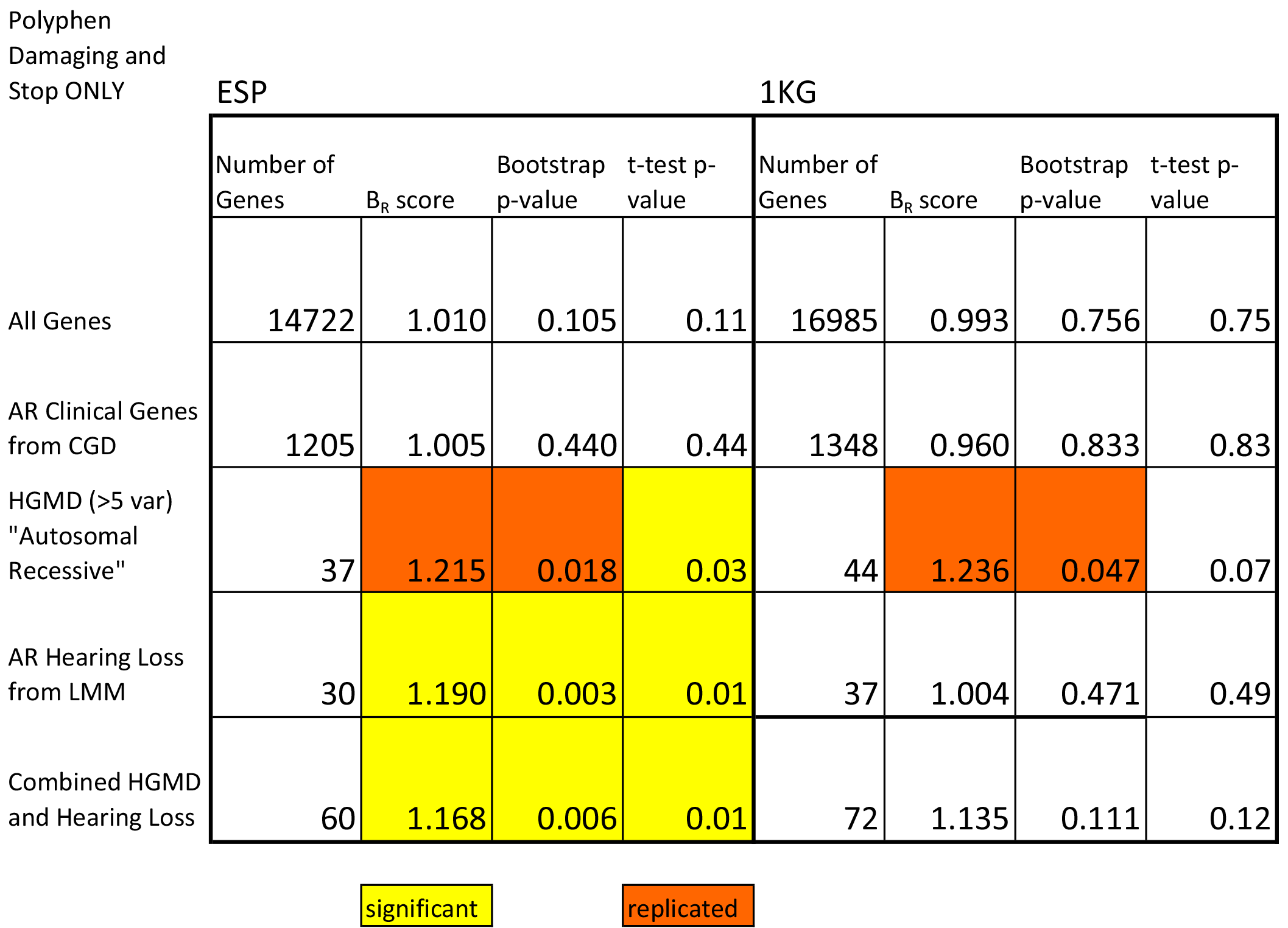

Supplement: S1 Table — Here we apply the B R statistic to sets consisting of genes known to be associated with autosomal recessive (AR) disease, as well as to a set of all genes in the genome. Only nonsense and human-free PolyPhen2 damaging variants are counted in these African and European population samples. Some results acquired from ESP data are replicated in 1KG, despite smaller population samples. For comparison, we display results of the paired Student t-test, which shows weaker ability to distinguish between distinct average mutation burdens in comparisons between African and European samples. (TIF) [file pgen.1005436.s007.tif]

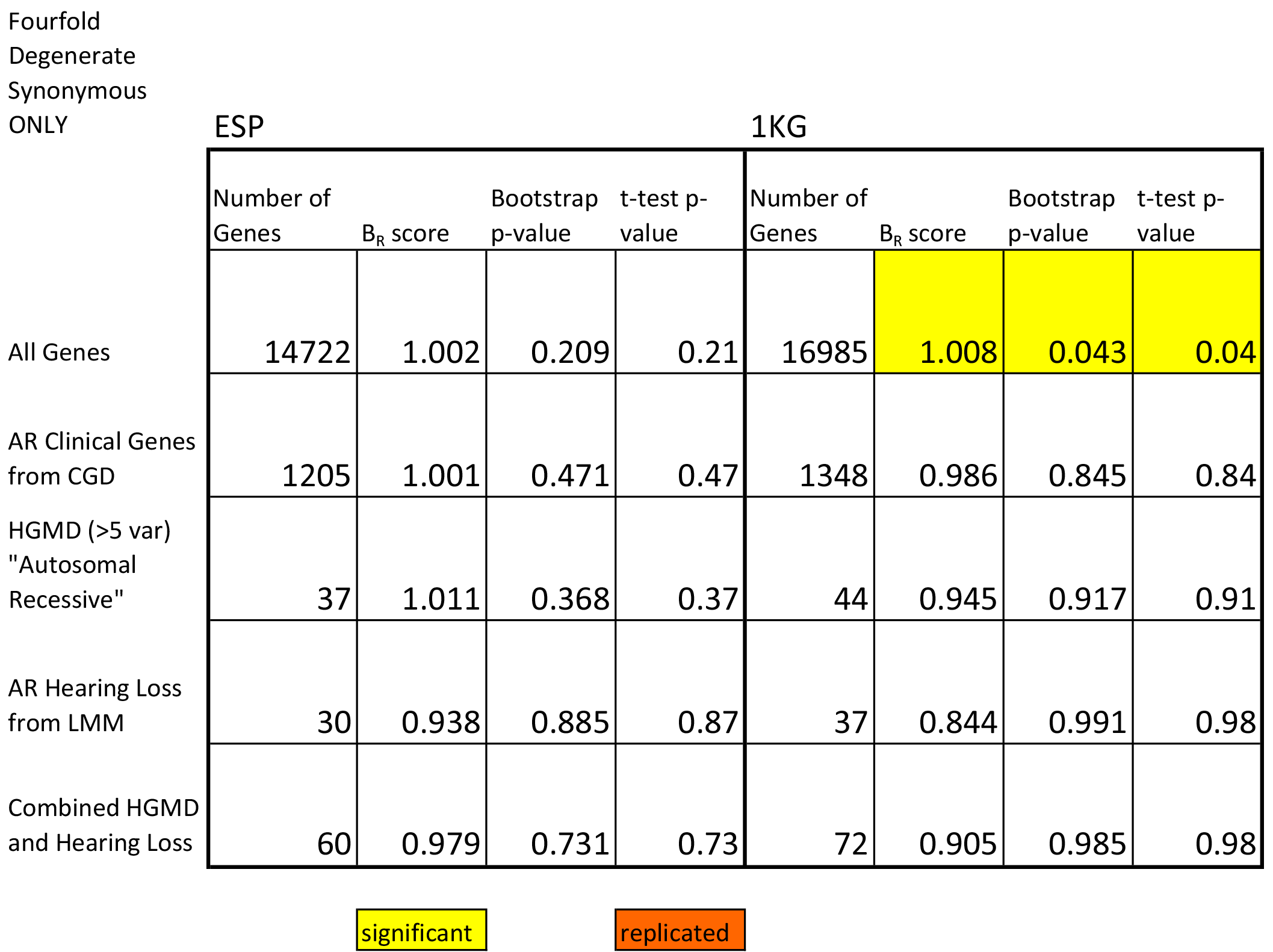

Supplement: S2 Table — All analyses are repeated for a B R statistic computed using only fourfold degenerate synonymous variants assumed to be under little or no selection. 1KG shows slight significance when testing all genes, however the value of B R remains very close to one, potentially indicating spurious significance. Naively, this provides a control for the results derived from nonsense and damaging variants above in the absence of selection or linkage to selected sites. (TIF) [file pgen.1005436.s008.tif]
